# Vorinostat Induces Apoptosis and Differentiation in Myeloid Malignancies: Genetic and Molecular Mechanisms

**DOI:** 10.1371/journal.pone.0053766

**Published:** 2013-01-08

**Authors:** Gabriela Silva, Bruno A. Cardoso, Hélio Belo, António Medina Almeida

**Affiliations:** 1 Unidade de Investigação em Patobiologia Molecular, Instituto Português de Oncologia de Lisboa Francisco Gentil, E.P.E., Lisboa, Portugal; 2 CEDOC, Faculdade de Ciências Médicas, Universidade Nova de Lisboa, Lisboa, Portugal; Wayne State University, United States of America

## Abstract

**Background:**

Aberrant epigenetic patterns are central in the pathogenesis of haematopoietic diseases such as myelodysplastic syndromes (MDS) and acute myeloid leukaemia (AML). Vorinostat is a HDACi which has produced responses in these disorders. The purpose of this study was to address the functional effects of vorinostat in leukemic cell lines and primary AML and MDS myeloid cells and to dissect the genetic and molecular mechanisms by which it exerts its action.

**Methodology/Principal Findings:**

Functional assays showed vorinostat promoted cell cycle arrest, inhibited growth, and induced apoptosis and differentiation of K562, HL60 and THP-1 and of CD33^+^ cells from AML and MDS patients. To explore the genetic mechanism for these effects, we quantified gene expression modulation by vorinostat in these cells. Vorinostat increased expression of genes down-regulated in MDS and/or AML (cFOS, COX2, IER3, p15, RAI3) and suppressed expression of genes over-expressed in these malignancies (AXL, c-MYC, Cyclin D1) and modulated cell cycle and apoptosis genes in a manner which would favor cell cycle arrest, differentiation, and apoptosis of neoplastic cells, consistent with the functional assays. Reporter assays showed transcriptional effect of vorinostat on some of these genes was mediated by proximal promoter elements in GC-rich regions. Vorinostat-modulated expression of some genes was potentiated by mithramycin A, a compound that interferes with SP1 binding to GC-rich DNA sequences, and siRNA-mediated SP1 reduction. ChIP assays revealed vorinostat inhibited DNA binding of SP1 to the proximal promoter regions of these genes. These results suggest vorinostat transcriptional action in some genes is regulated by proximal promoter GC-rich DNA sequences and by SP1.

**Conclusion:**

This study sheds light on the effects of vorinostat in AML and MDS and supports the implementation of clinical trials to explore the use of vorinostat in the treatment of these diseases.

## Introduction

Haematopoiesis is a complex, dynamic and controlled process where a pluripotent stem cell differentiates into different cell lineages. Growth factors, signaling transduction pathways and transcription factors regulate differentiation, cell cycle and apoptosis in these cells by modulating gene expression [1]. These processes are deregulated in haematopoietic diseases such as myelodysplastic syndromes (MDS) and acute myeloid leukemia (AML) [2], [3], [4].

MDS is characterized chiefly by increased apoptosis and progression to AML [2], whereas in AML there is a block in differentiation and increased cellular proliferation of neoplastic haematopoietic precursor cells [3], [4]. Knowledge of their pathophysiology has led to the development of novel treatment strategies which, unlike traditional cytotoxic therapies, use epigenetic agents to modulate gene expression.

Epigenetic modifications are important mechanisms by which cells regulate the expression of genes. Emerging data in the last decades revealed epigenetic changes play an important role in the pathogenesis of haematopoietic malignancies by silencing tumour suppressor genes and altering the expression of genes involved in a multitude of cellular functions [2], [3], [4], [5], [6], [7], [8]. Some of these epigenetic changes may be pharmacologically manipulated, such as with histone deacetylase inhibitors (HDACi), which are emerging as promising anti-cancer agents for the treatment of patients with MDS and AML [2], [3], [9], [10].

Vorinostat is a hydroxamic acid HDACi which promotes protein acetylation, modulates gene expression, and induces differentiation, growth arrest, and apoptosis of tumour cells [11], [12], and has shown promising clinical activity against haematological and solid tumours [10], [13], [14]. Clinical trials revealed vorinostat has only moderate haematologic toxicity and *in vitro* studies showed vorinostat exhibits low toxicity against normal cells [11], [12], [15]. Vorinostat anti-tumoural activity is believed to result from its ability to modulate gene expression, generate oxidative stress, and induce DNA damage and genomic instability [10], [16], [17].

The transcriptional effect of several HDACi on some genes is SP1 dependent [18], [19], [20], [21], [22], [23], [24], [25]. SP1 is a zinc finger transcription factor (TF) that regulates transcription of genes containing GC-rich DNA sequences in their promoters by modulating histone acetylation. SP1 regulates a variety of biological functions, including cell survival, growth, differentiation, and tumour development and progression [26].

Although the use of vorinostat for the treatment of haematological malignancies has increased substantially over the last years [10], the characterization of its effects on leukemic and MDS cells remains incomplete. The knowledge of the effects of vorinostat in these cells might contribute to a better understanding of its mechanisms of action, which may ultimately assist in its clinical application.

Our aim in this study was to document the functional effects of vorinostat in cell lines derived from patients with AML and primary AML and MDS myeloid cells and to contribute to the knowledge of the genetic and molecular mechanisms by which it exerts its action. Its functional effect was studied through cell cycle progression, apoptosis and differentiation assays. In an effort to explain the genetic mechanism by which these effects were obtained, we measured the modulation of expression of genes known to be involved in cell cycle regulation, apoptosis and oncogenesis, some of them known to be altered in MDS and/or AML [7], [8], [27], [28], [29], [30]. In an attempt to dissect the molecular mechanism of its action we analyzed the DNA sequence elements involved in vorinostat-mediated gene expression. In addition we analyzed vorinostat interaction with SP1.

## Materials and Methods

### Cell Lines, Patient Cells and Reagents

The human HL60 promyelocytic (AML M2/3, FAB classification), THP1 monocytic (AML M5) and erythroleukaemic K562 (myeloid blast crisis chronic myelogenous leukaemic cells with properties of AML M6) cells were cultured at 37°C and 5% CO2 in RPMI-1640 medium with 10% fetal calf serum, 2 mM L-glutamine, 100 µg/ml penicillin and 100 U/ml streptomycin (Life Technologies) in the presence of vorinostat (0.5–5 µM) or vehicle. Cells in logarithmic growth phase between passages 7 and 25 were used for these assays.

Peripheral blood (PB) samples from 8 patients with AML (3 monocytic (AML M5), 3 myelomonocytic (AML M4) , and 2 myeloblastic (AML M2)) and bone marrow (BM) aspirates from 9 patients with high risk MDS were obtained following informed consent and in the course of routine clinical workup. This project was approved by the local ethics committee and all samples were treated in accordance with institutional ethical regulations. Mononuclear cells (MNC) from PB and BM samples were separated by density gradient centrifugation through Ficcoll-Hypaque (Sigma) and CD33^+^ MNC isolated with human anti-CD33 microbeads (Miltenyi Biotec) as per manufactureŕs instructions. CD33^+^ cells (0.4–1×10^6^/ml) were cultured in RPMI-1640 or in Iscove’s Modified Dulbecco’s Medium (IMDM; Sigma) with 10% FCS, 2 mM L-glutamine and 100 µg/ml each penicillin and streptomycin (Life Technologies), 2 ng/ml SCF, 0.025 ng/ml GM-CSF, 0.025 ng/ml G-CSF, 0.1 ng/ml IL-3, 0.008 ng/ml IL-6 (Life Technologies) and 0.5 ng/ml EPO (Sigma) in the presence of vorinostat (0.5–5 µM) or vehicle.

Vorinostat (Selleck Chemicals), phorbol-12-myristate-13-acetate (PMA), and mithramycin A (Mith.A) (both from Sigma) were diluted in DMSO. Working solutions were prepared in PBS.

### Gene Expression Analysis (qPCR)

Total RNA was isolated from cells (0.7–1×10^6^/ml) cultured in complete RPMI plus vorinostat or vehicle (control) using the RNeasy Midi kit (Qiagen) according to the manufactureŕs instructions. cDNA was obtained from equal amount of purified RNA as described in [31]. Gene expression was quantified on Roche LightCycler 480 with gene specific primers for human Apoptosis and Cell cycle PCR Arrays (PAHS-3012G and PAHS-020F, SABiosciences). Results were normalized to HPRT1 mRNA in the same sample (or to B2M, HPRT1, RPL13A, and GAPDH mRNAs in the case of PCR Arrays) and calculated as fold change relative to control cells treated with vehicle. All experiments and analysis were carried out according to manufacturer’s instructions.

### Cell Cycle Analysis

Cell cycle progression was determined by flow cytometry using propidium iodide (PI). Cells (10^5^/ml) were cultured with increasing concentrations of vorinostat (1–5 µM) or vehicle (control) for 15, 24, 36 (all three cell lines) and 48 h (K562 and HL60), washed with PBS and fixed with ice-cold 70% ethanol. After 2 h, cells were washed twice in PBS and resuspended in PBS containing 50 µg/ml PI (Sigma), 200 µg/ml DNase free RNase A (Citomed), and 0,1% Triton X-100 for 1 h at room temperature. Acquisition was performed on a FACSCalibur flow cytometer (Becton Dickinson). Data were analyzed with the cell cycle program from FlowJo software (Tree Star, Inc. Ashland, OR).

### Cell Viability, Growth and Apoptosis

Cell viability was measured using the MTT (Sigma) colorimetric method. In short CD33^+^ cells were seeded in 96-well plates (100 µl/well) and exposed to varying concentrations of vorinostat. After 24–72 h treatment, 20 µl of MTT solution (20 mg/ml) was added to each well and cells incubated for 16 h. Thereafter, the resulting MTT crystals were dissolved in DMSO (120 µl/well) and absorbance measured at 490 nm using an ELISA microplate reader. Each concentration of the drugs was tested in triplicates. Cell growth was determined by microscopy using the trypan blue exclusion method. Apoptosis of cells co-stained with FITC-conjugated annexin-V (BD Biosciences) and PI or 7-AAD (BD Biosciences) was measured by flow cytometry as in [32].

### Flow Cytometry

Immunoflorescent staining was performed according to standard protocols. PE-CD33 antibody (BD biosciences) was used to assess CD33^+^ purity after positive selection. To assess vorinostat effect on differentiation of K562, HL60, cells (0.4–0.6×10^4^/ml) were cultured for 3 to 5 days in the presence of vorinostat (0.5–5 µM) or vehicle (control) in complete RPMI-medium in the presence or absence of GM/G-CSF or EPO as described elsewhere. To study differentiation of THP1 by vorinostat, cells (5×10^5^/ml) were cultured for 3 to 4 days in the presence of vorinostat (0.5–2 µM) or vehicle in complete RPMI-medium. Every 2 day half of the medium was replaced by fresh medium as above. PB-CD33 cells from AML patients and BM-CD33 cells from high risk MDS patients (0.4–0.6×10^4^/ml) were cultured in complete IMDM medium with SCF, IL-3, IL-6, GM-CSF, G-CSF, and EPO in the presence of 0.5–2 µM vorinostat or vehicle for 3 day. At day 3, 4 and 5 cells were harvested, stained and analyzed by flow cytometry.

Erythroid differentiation of K562 was assessed by co-staining cells with CD235A-PE (Beckman Coulter) and CD71-FITC (BD Biosciences) antibodies as in [33]. Myeloid differentiation of HL60 and CD33^+^ was evaluated with CD11b-PE plus CD13-APC, and CD11b-PE plus CD13-APC and CD14-FITC antibodies (Biolegend), respectively [34]. During neutrophil maturation four different stages of increasing maturation are defined based on the expression of CD13 and CD11b antigens. Stage I, CD13^hi^/CD11b^−^ myeloblasts; stage II, CD13^lo/int^/CD11b^−^ promyelocytes; stage III, CD13^lo/int^/CD11b^+^ myelocytes and metamyelocytes, and stage IV, CD13^hi^/CD1b^+^ bands/mature neutrophils. Monocytic maturation from monoblasts through promonocytes and monocytes is characterized by acquisition of CD11b and CD14. Most mature monocytic cells display bright CD14, CD11b and CD13 expression. Monocytic differentiation of THP1 cells was assessed by co-staining cells with CD11b-PE plus CD14-FITC antibodies. Acquisition was performed on a FACSCalibur and analysis with the FlowJo software. Specific labeling was compared with nonspecific staining using fluorescent-labeled isotype-matched control antibodies.

### K562 Cytological Staining and Hemoglobin (Hb) Determination

K562 cells (0.4–0.6×10^4^/ml) were cultured for 4 days at 37°C with 5% CO2 in RPMI-1640 medium with 10% fetal calf serum, 2 mM L-glutamine, 100 µg/ml penicillin and 100 U/ml streptomycin (Life Technologies) supplemented with 0.5 ng/ml EPO (Sigma) in the presence of vorinostat (1–5 µM) or vehicle. Every 2 day half of the medium was replaced by fresh medium as above. At day 4 after treatment, cells were harvested, washed with PBS, and vorinostat effect in terminal erythroid differentiation of K562 was assessed by measuring Hb content by ELISA and by microscopy of benzidine (to detect Hb) plus Giemsa stained cells. For benzidine–Giemsa staining, cells (2×10^3^ per culture) were centrifuged onto glass slides (2 min at 400 rpm), air dried, fixed (−20°C methanol) and stained with 3,3′-diaminobenzidine (TMB) and Giemsa stains according to the manufacturer’s recommendations (Sigma). Images were acquired on a Zeiss Axioplan microscope equipped with a Zeiss Axioxcam MRm (amplification: x200). For hemoblogin quantification by ELISA, the cell pellet was resuspended in lysis buffer (100 mM potassium phosphate pH 7.8, 0.2% Triton X-100) and incubated 10 min at room temperature. After pelleting cellular debris, the supernatant was collected and 20 µl was incubated with 80 µl TMB solution (2 mg/ml in 0.03% glacial acetic acid) containing H_2_O_2_ (0.03%). After 30 min incubation at room temperature in the dark, the enzymatic reaction was stopped by adding 50 µl of 1 N H_2_SO_4_ and absorbance was measured at 490 nm. A standard curve was generated by using known concentrations of human Hb (Sigma) as an internal control in each experiment. The amount of Hb in the cellular lysates was determined from the standard curve. After measurement of protein concentration of the lysate by the Bradford method, nanograms of Hb per micrograms of total cellular protein was calculated.

### Plasmids

DNA fragments containing the 2-1.5-kb proximal promoter and the 5′-UTR sequences of IER3, COX2 and cFOs genes were amplified from normal PB-MNC genomic DNA by PCR with promoter specific primers using the Expand Long Template PCR system (Roche) and cloned into the pGL3-basic plasmid (Promega) according to standard protocols. Progressive deletion reporter plasmids were generated by PCR using specific primers and the 2-1.5-kb reporter constructs. IER3 −61/+32 and −31/+32 reporter plasmids were generated by inserting oligonucleotides containing these promoter sequences into pGL3-basic plasmid. Reporter constructs with mutations in the putative TF binding sites of the IER3 promoter were generated using the −124/+32 IER3 reporter construct as template and mutagenic primers using the QuikChange site-directed mutagenesis kit (Stratagene). Reporter plasmids were amplified in Escherichia coli XL-2Blue (Promega) and DNA purified using DNA isolation kit (Qiagen). All reporter constructs were verified by sequencing.

### Reporter Assays

K562 and HL60 cells (10^5^ cells/ml well of 24-well plate) were transiently co-transfected with 400 ng indicated wild-type and mutated reporter constructs or with pGL3-basic control vector (Promega) plus 25 ng β-galactosidase reporter control expression vector (Promega) using 4 µl lipofectamine 2000 (Life Technologies) as per manufactureŕs protocol. 1 h after transfection cells were treated with 2 µM vorinostat or vehicle (control). 24 h later, cells were harvested, and luciferase and β-galactosidase activities of cell lysates measured using the Single Luciferase Assay System (Promega) and the Galacto-Light plus β-Galactosidase. Luciferase activities were normalized to β-galactosidase units in the same samples. Results are shown as average fold induction *versus* control cells transfected with pGL3 ± S.D. from one representative of at least three independent assays done in triplicate using each reporter construct at least from two different clones.

### Chromatin Immuneprecipitation (ChIP) Assays

K562 and HL60 cells (10^6^/ml) were treated with 5 µM vorinostat or vehicle for 7 h. Chromatin was cross-linked and sheared to 200–700 bp size according to the Shearing Chip Kit instructions (Diagenode) and immunoprecipitated with anti-SP1 polyclonal antibodies (Abcam ab13370 and Millipore 17–601) and isotype control IgG at 4°C during 16 h as described in the OneDay ChIP Kit protocol (Diagenode). After DNA recovery, the precipitates were evaluated by real time PCR with primers specific for the cFOS (−290/+8), COX2 (−356/−33), Cyclin G2 (−288/+15), IER3 (−100/+20), p21 (−174/+39), and CUL1 (−31/+41) promoter sequences. SP1 occupancy was calculated according to the OneDay ChIP Kit protocol. Results are expressed as fold change over control IgG and represent average values of at least three independent experiments ± SEM.

### SP1 Silencing and Western Blotting

K562 were transfected by electroporation with 100 nM of pooled SP1-specific or control siRNAs (L-026959-00-0005 and D-001210-03-05, respectively, Dharmacon) per 10^6^ cells as described in [35]. 4 h after transfection cells were treated with 2 µM vorinostat or vehicle (control). All assays were done in triplicate. SP1 knockdown was monitored 24 h thereafter by qPCR, and 48 and 72 h after transfection by Western blotting as described in [32]. SP1 protein was detected with mouse anti-human SP1 antibody (Santa Cruz Biotechnology sc-420X) and α-tubulin with a mouse anti-human α-tubulin monoclonal antibody (Sigma T9026). Primary antibodies were detected using horseradish peroxidase-conjugated goat anti-mouse IgG secondary antibody (Pierce). The experiments on SP1 knockdown effect on the expression of genes modulated by vorinostat were performed at 48 and 72 h after siRNA transfections by qPCR. Results were normalized to HPRT1 mRNA in the same sample and calculated as fold change over control cells treated with vehicle and transfected with the same siRNA.

### Bioinformatics

Gene sequences were obtained from GenBank and Ensembl databases. Analysis of promoter regions was performed with the Transcription Element Search System (TESS). Primers were designed with the Primer Express 3.0 software (ABI, Life Technologies), and NCBI primer express tool (http://www.ncbi.nlm.nih.gov/tools/primer-blast/). The sequences of all primers are available on request.

### Statistics

Statistical significance was determined using two-tailed Students t-test and the ANOVA and the Tukey-Kramer multiple comparison test. A value of p<0.05 was considered significant.

## Results

### Vorinostat Promotes Cell Cycle Arrest and Subsequent Apoptosis of K562, HL60, and THP1

**Figure 1:**
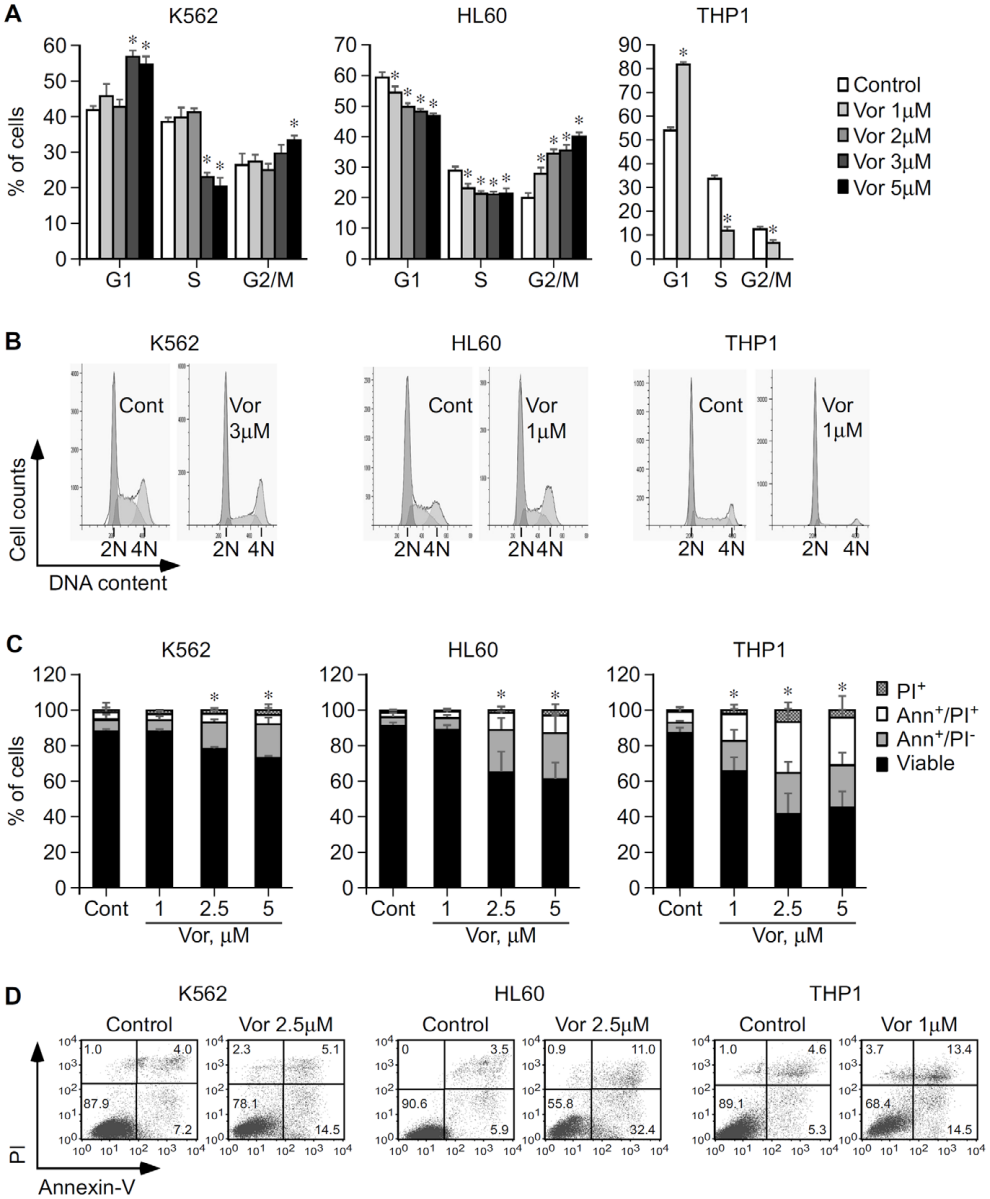
Functional impact of vorinostat in cell cycle progression and apoptosis of K562, HL60, and THP1 cells. **A,** K562, HL60 and THP1 cells were cultured with vorinostat or vehicle (Control/Cont) as indicated and cells were analyzed by flow cytometry after 15 h (K562 and HL60) and 36 h (THP1). Graphs show average percentage of cells in each phase of the cell cycle ± SD of three independent assays for K562 and HL60 and two different experiments for THP1, each in triplicate. B, Representative histograms showing the effect of vorinostat in K562, HL60 and THP1 cell cycle progression. Cells arrested in the G1 phase, 2 N DNA content; cells arrested in S phase; and cells arrested in the G2/M phase (4 N DNA content). C, K562, HL60 and THP1 cells were cultured in the presence of vorinostat or vehicle as indicated and apoptosis was analyzed 48 h thereafter by flow cytometry. Graphs show average percentage of K562, HL60 and THP1 apoptosis ± SD of three independent assays, done in triplicate. D, Representative dot plots showing the percentage of apoptotic K562, HL60 and THP1 cells cultured for 48 h in the absence and in the presence of vorinostat. Numbers are percentages of total cells in the respective gates from one of three independent experiments for each cell line. *p<0.05.

In order to be effective in MDS and AML, vorinostat would have to induce cell cycle arrest and commit neoplastic cells to apoptosis. This hypothesis was tested by flow cytometric analysis of cell cycle and apoptosis of leukemic K562, HL60, and THP1 cells treated with vorinostat. Our results revealed that vorinostat induced cell cycle arrest of K562, HL60 and THP1 cell lines. In K562 incubation with 3–5 µM vorinostat for 15–24 h caused G1 and G2/M arrest accompanied by a significant reduction of cells in the S phase and a subsequent accumulation of cells in the sub-G1 population (Figure 1A and B and Figure S1A and S1B). In HL60 15 h exposure to vorinostat caused significant G2/M arrest and reduction of cells in the G1 and S phases in a dose dependent manner (Figure 1A and B) with later accumulation in the sub-G1 population (data not shown). Treatment of THP1 cells with 1 µM vorinostat for at least 24 h caused substantial G1 arrest with significant reduction of cells in the S and G2/M phases of the cell cycle (Figure 1A and B).

**Figure 2:**
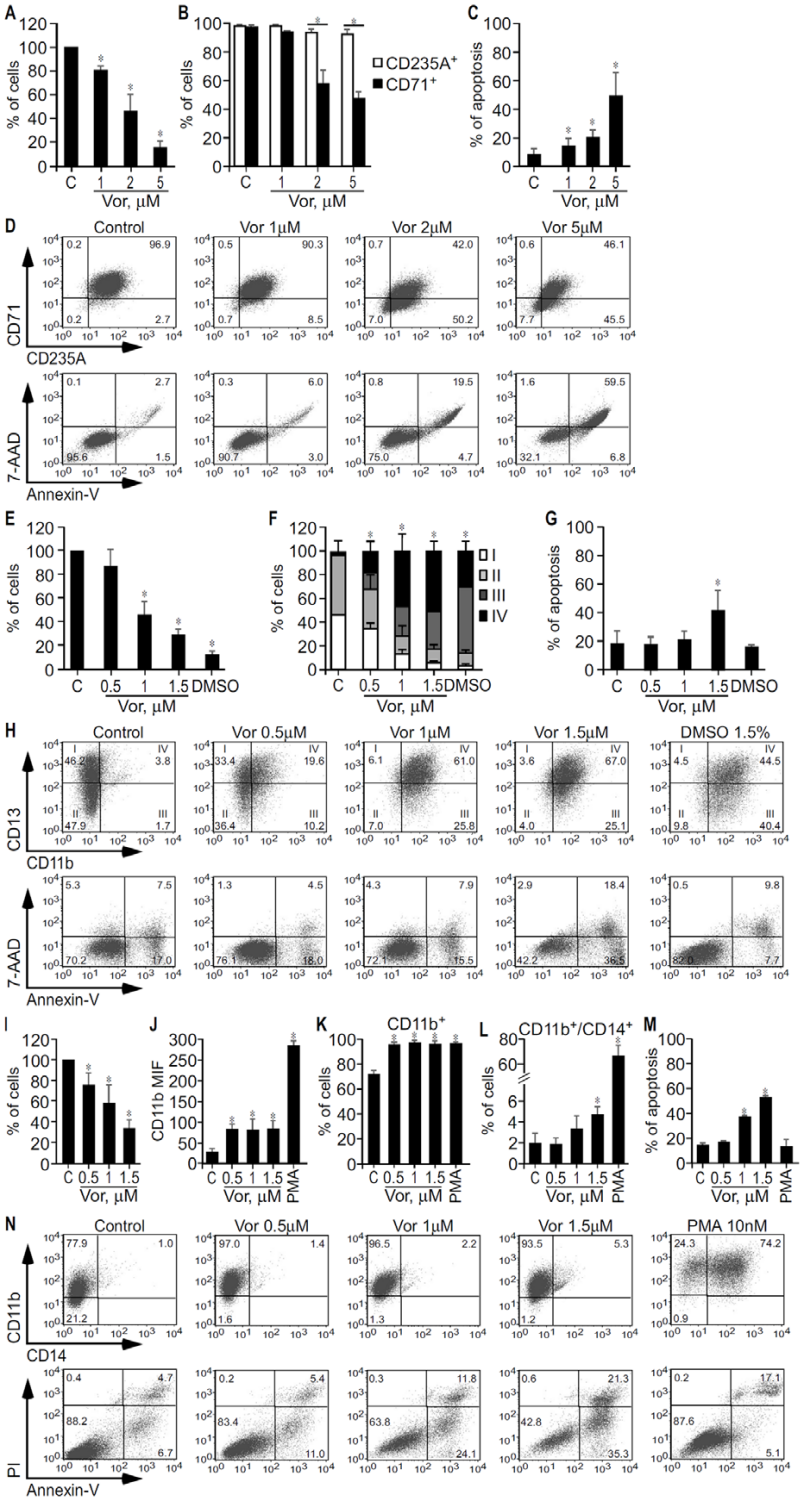
Effect of vorinostat on growth and differentiation of K562, HL60 and THP1 cells. K562 (A–D), HL60 (E–H) and THP1 (I–N) cells were treated with vorinostat as indicated, vehicle (Control/C), DMSO (1.5%) or PMA (10 nM). After 3 days, K562, HL60 and THP1 cellular growth was determined by trypan blue exclusion method, and surface markers and apoptosis by flow cytometry. A, Average percentage of K562 cells ± SD of four independent experiments, done in duplicate. B, Average percentage of K562 cells expressing the erythroid CD235A marker and the transferin CD71 receptor ± SD of three independent experiments, done in triplicate. C, Average percentage of annexin-V stained apoptotic cells ± SD obtained in the three experiments shown in (B). D, Representative dot blots showing the expression profile of CD235A and CD71 markers and apoptosis in K562 cells treated with vehicle and increasing concentrations of vorinostat. E, Average percentage of HL60 cells ± SD of four independent experiments, done in duplicate. F, Average percentage of HL60 cells on different myeloid maturation stages according the CD13/CD11b expression profile ± SD of four independent experiments, done in triplicate. I (CD13^hi^/CD11b^−^): myeloblasts; II (CD13^lo/int^/CD11b^−^): promyelocytes; III (CD13^lo/int^/CD11b^+^): myelocytes and metamyelocytes; and IV (CD13^hi^/CD11b^+^): band cells and mature neutrophils. G, Average percentage of annexin-V stained apoptotic cells ± SD obtained in the four experiments shown in (F). H, Representative dot blots showing the expression profile of CD11b and CD13 antigens and apoptosis in HL60 treated with vehicle, vorinostat, and DMSO. Numbers in CD13/CD11b plots are percentage of CD33 cells on different myeloid maturation stages. Numbers in other panels are percentages of total cells in the respective gates. I, Average percentage of THP1 cells ± SD of three independent experiments, done in duplicate or triplicate. J, Median fluorescent intensity of CD11b monocytic differentiation marker expressed on the cell surface of THP1 cells. K, Percentage of THP1 cells expressing CD11b. L, Percentage of THP1 cells double positive for CD11b and CD14. Values in (J, K and L) are average values ± SD of three independent experiments, each done in triplicate. M, Average percentage of annexin-V stained apoptotic cells ± SD obtained in the three experiments shown in (J, K and L). N, Representative dot blots showing the expression profile of CD11b and CD14 markers and apoptosis in THP1 cells treated with vehicle, vorinostat and PMA. *, p<0.05.

The significant accumulation of cells in the sub-G1 population suggests these were undergoing apoptosis. This was verified by flow cytometry by staining treated cells with annexin-V and PI/7-AAD. No significant apoptosis was observed until at least 24 h post-treatment. At 48 h, 2.5 µM vorinostat induced significant apoptosis in K562 (P = 0.03) and HL60 (P<0.001) (Figure 1C and D), consistent with the accumulation of cells in sub-G1 at this time point. After 72 h treatment, lower (1.5–2 µM) vorinostat concentrations induced significant apoptosis of K562 and HL60 cells (Figure 2C and G, and Figure S1C and S1D). In both cases, vorinostat induced considerably more apoptosis in HL60 than in K562. In THP1 48 h treatment with 1 µM vorinostat induced substantial apoptosis (P = 0.0001) (Figure 1C and D). THP1 cells were those most sensitive to the apoptotic effects of vorinostat.

Overall these results show that vorinostat induces cell cycle arrest and apoptosis of K562, HL60 and THP1. The three cell lines have different sensitivities to vorinostat, HL60 and THP1 being substantially more sensitive to vorinostat-mediated cell cycle arrest and apoptosis than K562.

### Vorinostat Inhibits Growth and Promotes Differentiation of K562, HL60, and THP1

Given that increased proliferation and differentiation block are hallmark features of MDS and AML [2], [3], we analysed the effect of vorinostat on these cell lines’ growth and differentiation, as determined by trypan blue exclusion by microscopy and flow cytometry. As shown in Figure 2, after 3 days of treatment, vorinostat inhibited growth (Figure 2A) and promoted erythroid differentiation of K562, as reflected by the increase of more mature CD235A^+^/CD71^−^ cells from 3% to 50% (Figure 2B and D), and induced apoptosis (Figure 2C and D). However, vorinostat did not increase hemoglobin content as detected by both benzidine–Giemsa stain and ELISA in K562 cells at the end of 3 to 5 days treatment (Figure S2). Significantly, this effect was identical to that obtained with EPO (data not shown).

Myeloid maturation in HL60 was based on the identification of four different stages of increasing maturation, defined according to the expression profile of CD13 and CD11b [34], as determined by flow cytometry. As shown in Figure 2E–H, vorinostat inhibited growth (Figure 2E) and promoted HL60 terminal differentiation in a dose dependent manner (P<0.05) (Figure 2F and H). Maximal HL60 terminal myeloid maturation is observed at vorinostat concentrations that substantially inhibit growth and induce apoptosis. When treated with 1–1.5 µM vorinostat, HL60 cells that are composed by myeloblasts (stage I cells) and promyelocytes (stage II cells), are composed by myelocytes/metamyelocytes (stage III cells) and mostly by bands/mature neutrophils (stage IV cells). DMSO was used as a positive control [36] and GM-CSF plus M-CSF produced identical results (data not shown).

THP-1 cell line is composed by monoblasts and promonocytes committed to the monocytic cell lineage [37]. Monocytic differentiation of THP1 cells was determined according to the expression of CD11b and CD14 by flow cytometry. PMA was used as positive control. As shown in Figure 2I–N, following 3 days’ incubation, vorinostat (0.5–1.5 µM) inhibited growth and induced apoptosis of THP1 in a dose dependent manner and promoted their differentiation as reflected by increased expression of the monocytic differentiation marker CD11b and increased percentage of CD11b^+^ cells. THP1 cells treated with 0.5 µM vorinostat, a concentration that caused significant growth inhibition but not apoptosis, increased the expression of CD11b by 2.5 fold *versus* control cells and the percentage of CD11b^+^ cells from 75% to almost 100% (Figure 2J, K and N). At 1.5 µM, a concentration that caused considerable growth arrest and apoptosis, vorinostat induced a greater degree of THP1 differentiation, as reflected by the simultaneous expression of CD11b and CD14, a marker of mature monocytes (Figure 2L–N).

Overall this series of results show vorinostat inhibits cell proliferation and promotes differentiation of K562, HL60 and THP1. The sensitivities of the different cell lines to vorinostat varied, with a greater degree of differentiation but also more apoptosis seen in HL60 and THP1.

### Vorinostat Induces Apoptosis and Promotes Differentiation of AML and MDS CD33^+^ Cells

**Figure 3:**
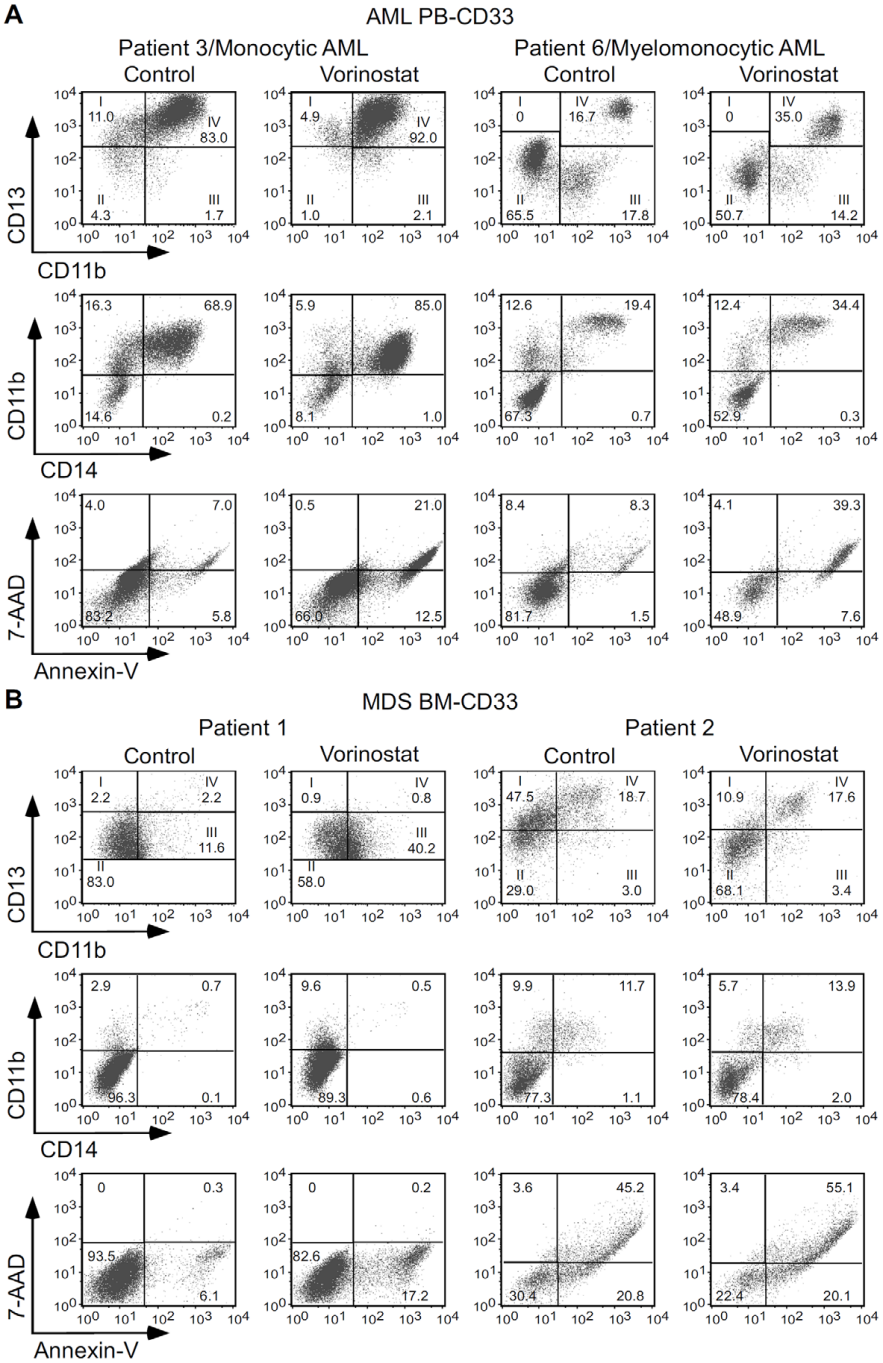
Effect of vorinostat on myeloid differentiation and apoptosis of CD33^+^ cells from AML and AML patients. CD33^+^ cells from peripheral blood of AML patients (A) and bone marrows of MDS patients (B) were cultured in complete IMDM medium with SCF, IL-3, IL-6, GM-CSF, G-CSF, and EPO in the presence of 1 µM vorinostat or vehicle (Control). After 3 days, myelomonocytic markers, CD11b, CD13 and CD14 and apoptosis were analyzed by flow cytometry. The numbers in CD13/CD11b panels are percentage of CD33 cells on different myeloid maturation stages according the CD13 and CD11b expression levels. I (CD13^hi^/CD11b^−^): myeloblasts; II (CD13^lo/int^/CD11b^−^): promyelocytes; III (CD13^lo/int^/CD11b^+^): myelocytes and metamyelocytes; and IV (CD13^hi^/CD11b^+^): band cells and mature neutrophils. Mature monocytes are also CD13^hi^/CD11b^+^. Numbers in other panels are percentages of total cells in the respective gates.

The effects of vorinostat observed in HL60 and THP1 cells were confirmed in CD33^+^ cells isolated from blood of patients with AML (AML PB-CD33). Myeloid and monocytic differentiation was determined according to the expression of CD13, CD11b and CD14 by flow cytometry. Following 3 days’ incubation, vorinostat (0.5–1 µM) induced differentiation of these cells as demonstrated by increased proportions of more mature CD13/CD11b myeloid populations (CD13^lo/int^/CD11b^+^, and mostly CD13^hi^/CD11b^+^) and decreased proportions of the two more immature CD13/CD11b populations (CD13^hi^/CD11b^−^ and CD13^lo/int^/CD11b^−^), and also by increased percentage of mature monocytes, i.e. CD14^+^/CD11b^+^ cells (Table 1 and Figure 3A). As in the case of leukaemic cell lines, vorinostat also significantly increased apoptosis, suggesting these two events are correlated.

**Table 1:** Vorinostat effect in myeloid differentiation of CD33^+^ cells from AML patients.

		CD13/CD11b				CD14/CD11b				
		CD13^hi^ /CD11b^−^	CD13^lo/int^ /CD11b^−^	CD13^lo/int^ /CD11b^+^	CD13^hi^ /CD11b^+^	CD14^−^ /CD11b^−^	CD14^−^ /CD11b^+^	CD14^+^ /CD11b^+^	Apoptosis	Viability
		Stage I	Stage II	Stage III	Stage IV					
Patient 1	Control	1.3	43.4	4.7	49.6	nd	nd	nd	nd	100
Monocytic AML	Vorinostat	0.8	29.6	7.8	61.2	nd	nd	nd	nd	84.4
Patient 2	Control	16.1	63.7	9.2	10.7	nd	nd	nd	nd	100
Monocytic AML	Vorinostat	13.4	45.7	26.9	13.7	nd	nd	nd	nd	87.6
Patient 3	Control	11.0	4.3	1.7	83.0	14.6	16.3	68.9	12.8	100
Monocytic AML	Vorinostat	4.9	1.0	2.1	92.0	8.1	5.9	85.0	33.5	83
Patient 4	Control	7.8	12.7	6.9	71.4	28.0	25.6	45.1	7.4	nd
Myeloblastic AML	Vorinostat	6.1	9.8	6.4	77.4	22.5	10.4	65.7	13.7	nd
Patient 5	Control	2.4	56.7	6.1	34.6	59.0	5.6	34.7	3.3	nd
Myelomonocytic AML	Vorinostat	1.1	50.2	5.6	42.5	51.1	2.8	45.6	13.0	nd
Patient 6	Control	0.02	65.5	17.8	16.7	67.3	12.6	19.4	9.8	nd
Myelomonocytic AML	Vorinostat	0.02	50.7	14.2	35.0	52.9	12.4	34.4	46.9	nd
Patient 7	Control	1.1	9.7	9.0	80.2	10.8	17.1	71.9	15.6	nd
Myelomonocytic AML	Vorinostat	0.1	4.1	11.0	84.8	4.3	14.3	81.4	27.2	nd

Peripheral blood CD33^+^ cells from AML patients were cultured ex-*vivo* in the presence of 1 µM vorinostat or vehicle (control) and myeloid differentiation analyzed by flow cytometry with CD11b-PE plus CD13-APC or CD11b-PE plus CD13-APC and CD14-FITC antibodies. Cellular apoptosis was assessed by flow cytometry of annexin-V-FITC/7-AAD co-stained cells and viability by the MTT method. Results are percentage of cells per population subset. nd, not determined.

Since epigenetic changes are implicated in both AML and MDS and a significant proportion of MDS patients transform to AML [2], [3], [8], we evaluated whether vorinostat promoted differentiation of myeloid cells from MDS patients. As shown in Figure 3B, vorinostat induced similar differentiation effects in CD33^+^ cells isolated from bone marrows of patients with high risk MDS (MDS BM-CD33). Vorinostat (1 µM) promoted a 3 fold increase of the percentage of stage III cells in patient 1, and a 2 fold increase of stage II cells in patient 2, and induced apoptosis.

### Vorinostat Modulates the Expression of Genes Involved in Cell Cycle Control, Proliferation, Apoptosis, and Differentiation of Haematopoietic Cell Lines

**Figure 4:**
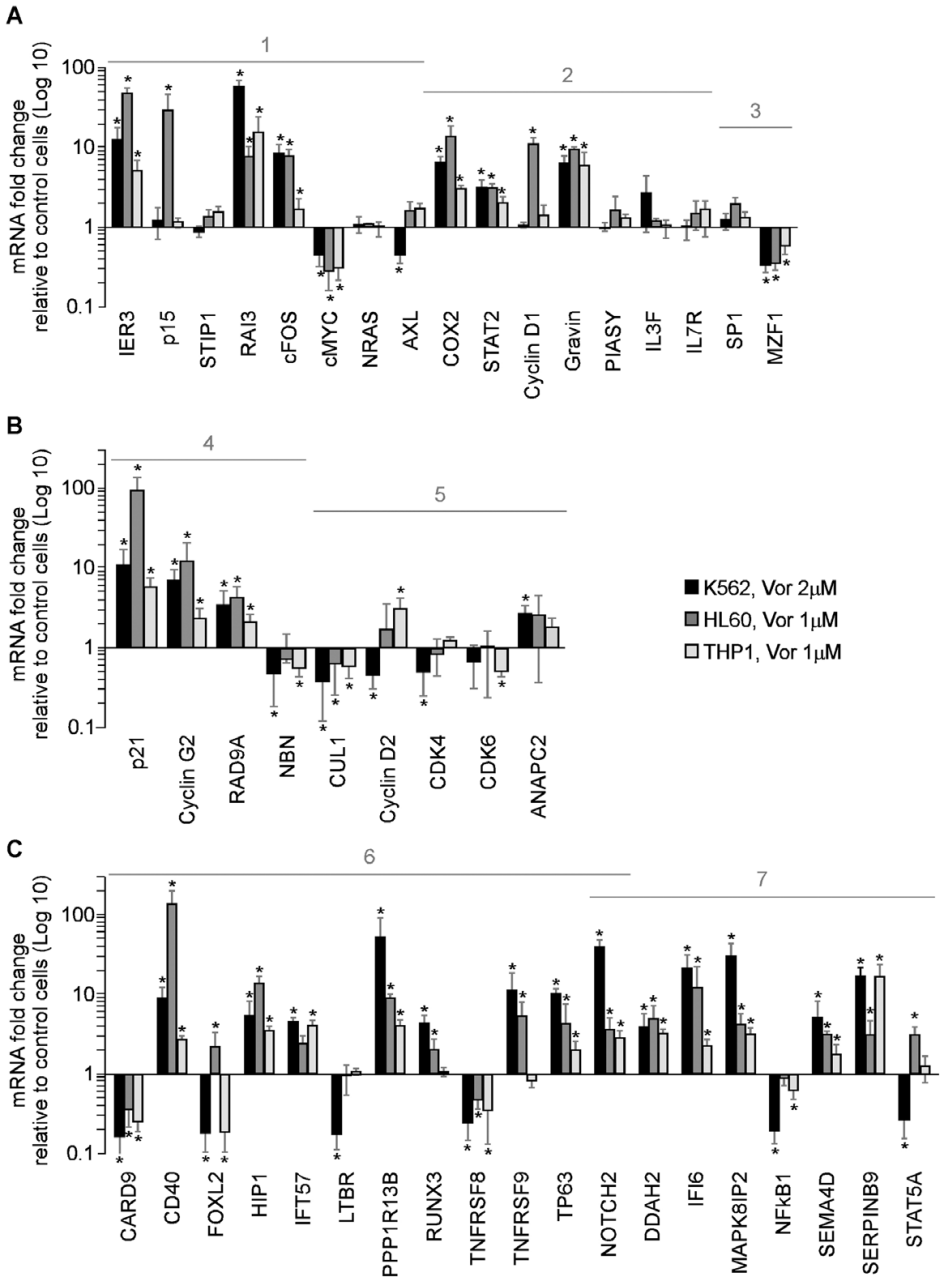
Effect of vorinostat on genes involved in regulation of cell cycle control, proliferation, apoptosis, and differentiation in K562, HL60 and THP1 cells. K562 HL60, and THP1 cells were treated with vorinostat as indicated or vehicle (Control) for 4 h and gene expression quantified by qPCR. A, Effect of vorinostat on the expression of: 1- genes with altered expression in haematologic malignancies; 2- genes with altered expression in haematologic malignancies known to respond to epigenetic therapy; 3- transcription factors. B, Effect of vorinostat on the expression of genes that control: 4- cell cycle arrest/check point/DNA repair and 5- cell cycle transition. C, Vorinostat effect on the expression of: 6- pro-apoptotic and 7-anti-apoptotic genes. Results are represented as average values ± SD from at least four independent assays, performed in triplicate, for both cell lines. *p<0.05.

In order to understand the mechanisms through which vorinostat may exert its functional effects, we first assessed the effect of vorinostat on the expression of the following groups of genes:

1. cell cycle control, proliferation, apoptosis, differentiation
  - altered in MDS and/or AML [7], [8], [27], [28], [29], [30] (Figure 4A, 1);
  - altered in MDS and/or AML and known to be modulated by epigenetic agents [7], [8], [27], [28], [29] (Figure 4A, 2);
2. transcription factors involved in oncogenesis [26], [38] (Figure 4A, 3).

In K562, HL60 and THP1 vorinostat (1–2 µM/8 h) significantly modulated the expression of genes involved in cell cycle control, proliferation, apoptosis, differentiation and oncogenesis. Specifically, IER3, RAI3, cFOS, COX2, STAT2 and Gravin, frequently suppressed in MDS patients [7], [8], were significantly up-regulated by vorinostat in both cell lines (Figure 4A, 1 and 2), whereas c-MYC, a major player in haematopoiesis and often deregulated in many haematological disorders [39], and MZF1, involved in oncogenesis progression [38], were significantly down-regulated (Figure 4A, 1 and 3). In THP1 the effect of vorinostat on the expression of most of these genes was less pronounced than in K562 and HL60. Interestingly, some genes, such as p15 (Figure 4A, 1), and Cyclin D1 (Figure 4A, 2), often deregulated in MDS and AML and associated with worse prognosis [7], [40], [41], [42], were modulated to a greater extent in HL60, whereas AXL (Figure 4A, 1), frequently up-regulated in AML and associated with adverse prognosis [30], was more marked in K562.

**Figure 5:**
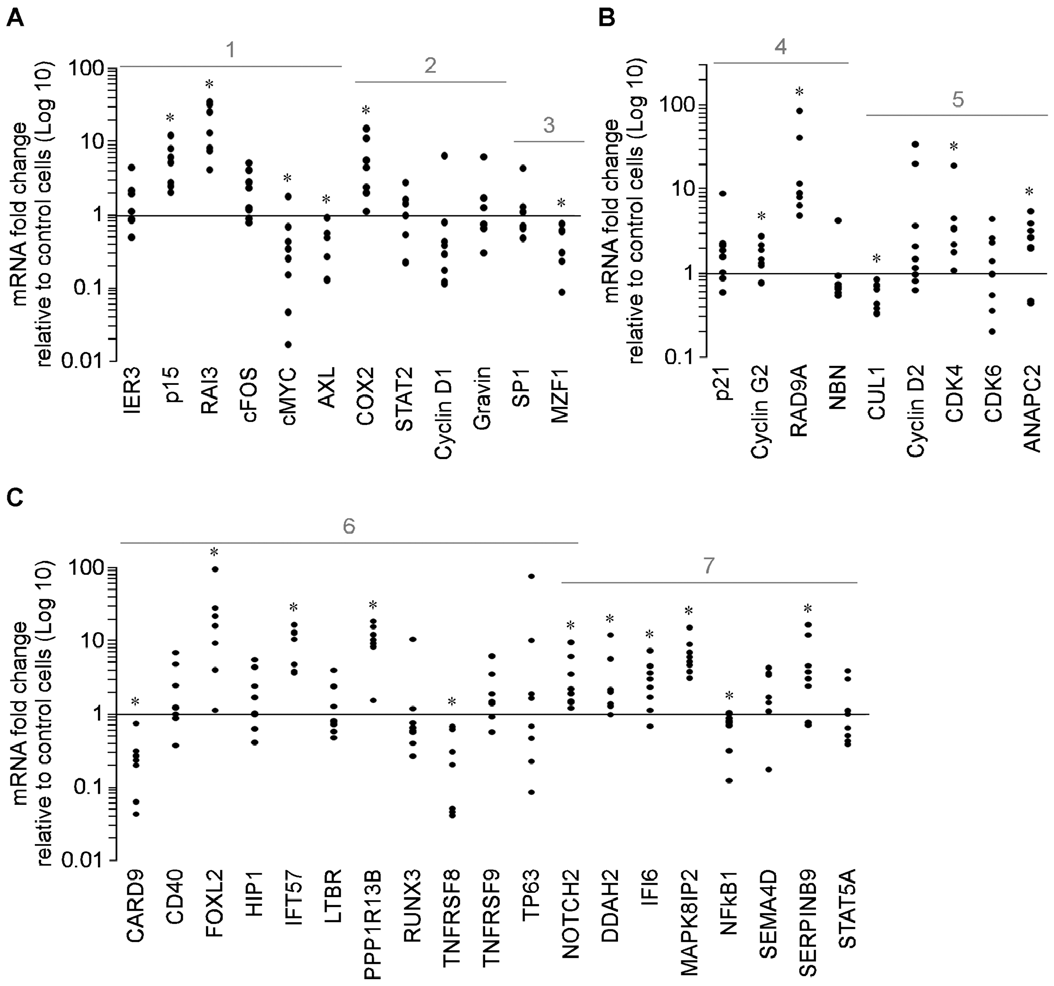
Effect of vorinostat on genes involved in regulation of cell cycle control, proliferation, apoptosis, and differentiation in CD33 myeloid cells from AML patients. PB-CD33 cells from AML patients were treated with 5 µM vorinostat or vehicle (Control) for 8 h and gene expression quantified by qPCR. A, Effect of vorinostat on the expression of genes responsive to vorinostat in K562, HL60 and THP1 cells; 1- genes with altered expression in haematologic malignancies; 2- genes with altered expression in haematologic malignancies that respond to epigenetic therapy; and 3- transcription factors. B, Effect of vorinostat on the expression of genes that control: 4- cell cycle arrest/check point/DNA repair and 5- cell cycle transition. C, Effect of vorinostat on the expression of: 6- pro-apoptotic and 7-anti-apoptotic genes. In all panels, each dot represents data from one patient. *p<0.05.

Using cell cycle and apoptosis PCR arrays, we next assessed the effect of vorinostat in other cell cycle and apoptosis genes, as these genes are key players in haematological malignancies [43]. We found that, in K562, vorinostat induced a 4 fold or greater change in 9 cell cycle and 21 apoptosis genes. These findings were confirmed in K562, HL60 and THP1 cells by qPCR (Figure 4B and C). Vorinostat induced transcription of genes involved in cell cycle arrest (p21 and Cyclin G2) [44] and DNA repair (RAD9A) [45] and decreased the expression of NBN, a double strand break repair gene in all cell lines (Figure 4B, 4). Up-regulation of cell cycle arrest genes by vorinostat was more marked in HL60. Down-regulation of cell cycle transition genes was more marked in K562 than in THP1 and HL60 (Figure 4B, 5).

**Figure 6:**
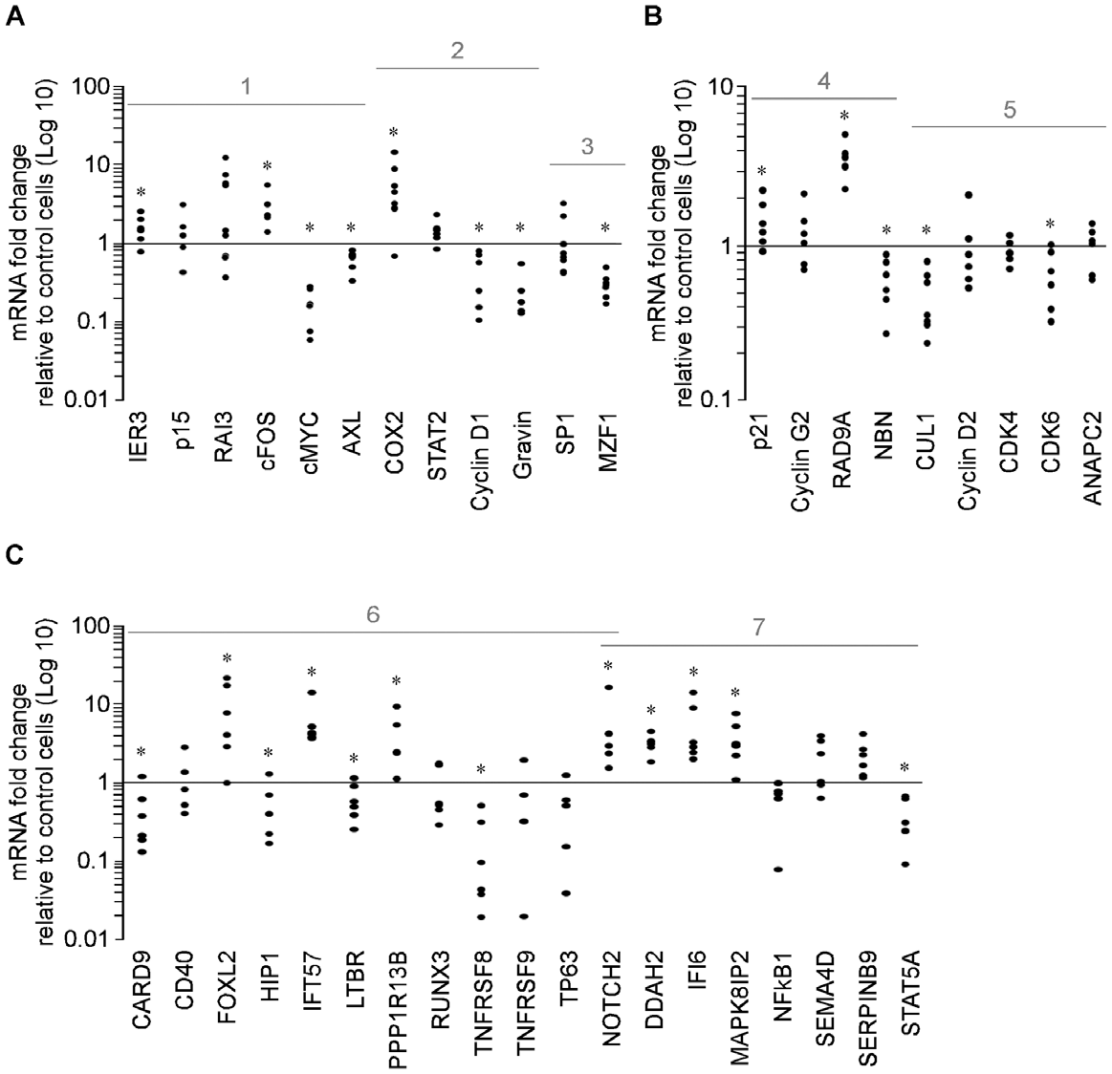
Effect of vorinostat on genes involved in regulation of cell cycle control, proliferation, apoptosis, and differentiation in CD33 myeloid cells from MDS patients. BM-CD33 cells from MDS patients were treated with 5 µM vorinostat or vehicle (Control) for 8 h and gene expression quantified by qPCR. A, Effect of vorinostat on the expression of genes responsive to vorinostat in K562, HL60 and THP1; 1- genes with altered expression in haematologic malignancies; 2- genes with altered expression in haematologic malignancies that respond to epigenetic therapy; and 3- transcription factors. B, Effect of vorinostat on the expression of genes that control: 4- cell cycle arrest/check point/DNA repair and 5- cell cycle transition. C, Effect of vorinostat on the expression of: 6- pro-apoptotic and 7-anti-apoptotic genes. In all panels, each dot represents data from one patient. *p<0.05.

**Figure 7:**
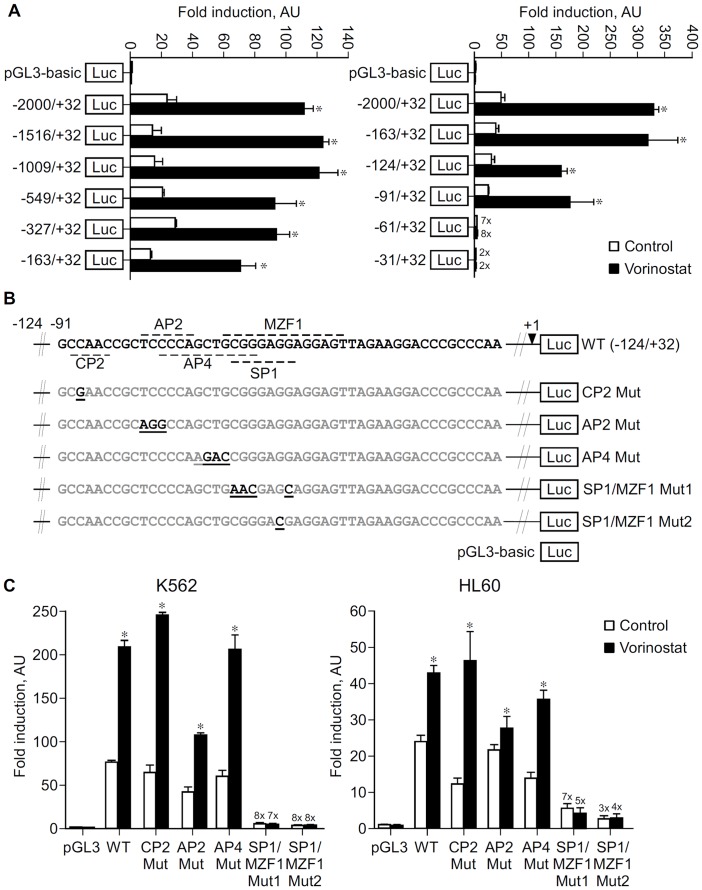
Identification of vorinostat responsive elements in the IER3 promoter. A, K562 cells were transfected with pGL3-basic vector or a series of IER3 reporters as indicated plus β-galactosidase vector, and then treated with vorinostat (2 µM) or vehicle (Control) for 24 h and reporter activity measured. Results are shown as average fold luciferase/β-galactosidase induction *versus* control cells transfected with pGL3 ± S.D. from one representative of at least three independent assays done in triplicate using each reporter construct at least from two different clones. B, Scheme of wild type and mutated −124/+32 reporter constructs showing the −91 to −43 nucleotide sequence of IER3 promoter containing the putative TF binding sites present in the −91/−61 region (lane 1) and the mutated nucleotides for the different TF binding sites present in this region (lanes 2 to 6). The mutated nucleotides present in the mutant luciferase reporter plasmids for the different TF binding sites are boldfaced and underlined. +1, denotes transcription start site. C, K562 and HL60 cells were transfected with pGL3-basic vector or with wild type and mutated −124/+32 IER3 reporter constructs for the indicated TF plus β-galactosidase vector and the rest of the procedure was done as in (A).The results are average fold luciferase/β-galactosidase induction versus control cells transfected with pGL3 ± S.D. from one representative assay done in triplicate using mutated plasmids for the same putative TF binding site from different clones, of at least three independent assays performed in both K562 and HL60 cells. Data were analyzed using the ANOVA and the Tukey-Kramer multiple comparison test. *p<0.05.

The effect of vorinostat on the expression of apoptosis genes (all genes in Figure 4C, p21 in Figure 4B, and IER3 in Figure 4A) differed between the three cell lines: in K562 15 apoptosis genes were up-regulated and 6 down-regulated whereas in HL60 cells 16 apoptosis genes were up-regulated and 2 down-regulated. In THP1 13 apoptosis genes were up-regulated and 3 down-regulated, showing that modulation of apoptosis genes by vorinostat was relatively lower in THP1 cells when compared to the other cell lines. In all cell lines vorinostat significantly increased the transcription of the pro-apoptotic genes CD40, HIP1, PPP1R13B, TP63 and NOTCH2 , did not affect expression of IFT57 and LTBR in HL60 and LTBR, RUNX3 and TNFRSF9 in THP1 (Figure 4C, 6). Vorinostat suppressed the pro-apoptotic genes CARD9 and TNFRSF8 in the three cell lines, FOXL2 in K562 and THP1 cells, and LTBR only in K562 (Figure 4C, 6). The anti-apoptotic genes up-regulated by vorinostat in the three cell lines included genes coding for caspase inhibitors (i.e. IFI6 and SERPINB9) and proteins involved in signal transduction pathways with multifaceted functions (i.e. DDAH2, MAPK8IP2, SEMA4D) [46], [47] (Figure 4C, 7). Of note, NFκB1 was suppressed in K562 and THP1 cell lines and STAT5A only in K562.

Collectively, these data demonstrate that, at clinically relevant concentrations (i.e. 1 and 2 µM) [14], [48], vorinostat modulates gene expression in K562, HL60, and THP1 cells in a manner consistent with the promotion of cell cycle arrest, differentiation and apoptosis. Of note, similar gene expression profiles were obtained with 5 µM vorinostat in K562 and HL60 cells after 8 h incubation (data not shown).

### Vorinostat Modulates the Expression of Genes Involved in Cell Cycle Control, Proliferation, Apoptosis, and Differentiation in Primary Myeloid Cells from AML and MDS Patients

These profiles of modulation of gene expression by vorinostat were confirmed in PB-CD33 cells from AML patients with circulating blasts. As shown in Figure 5A, the effect of vorinostat (5 µM/8 h) on the expression pattern of genes altered in MDS and/or AML, with the exceptions of STAT2, Gravin and Cyclin D1, mirrored that observed in K562, HL60, and THP1 cell lines: p15, RAI3 and COX2 were significantly up-regulated, IER3 and cFOS moderately up-regulated, c-MYC, AXL and MZF1 suppressed. Interestingly, Cyclin D1, which is induced by vorinostat in HL60, is significantly down-regulated by this agent in primary AML cells. The modulation of expression of STAT2 and Gravin was not consistent between different patients (Figure 5A, 2).

In AML PB-CD33 cells, the effect of vorinostat on the expression of genes involved in cell cycle regulation also mirrored that seen in cell lines, albeit to a lesser extent. p21, Cyclin G2, RAD9A and ANAPC2 were induced whereas NBN and CUL1 were suppressed. Exception was CDK4 that was significantly up-regulated in these cells (Figure 5B) contrary to the cell lines where it was either suppressed (K562) or unaffected (THP1 and HL60). In primary cells, vorinostat substantially increased transcription of the pro-apoptotic caspase activating genes FOXL2, unlike that seen in K562 and THP1 and induced considerable higher levels of IFT57 as compared to the cell lines. In primary cells there was no consistent effect on the expression of CD40, HIP1, LTBR, RUNX3, TNFRSF9 and TP63 by vorinostat (Figure 5C, 6). As in the case of K562, HL60 and THP1, CARD9 and TNFRSF8 were suppressed.

Vorinostat significantly up-regulated NOTCH2 and anti-apoptotic genes DDAH2, IFI6, MAPK8IP2, SEMA4D, SERPINB9 and down-regulated NFκB1 in primary cells similarly to that seen with the K562 and THP1 cell lines. No consistent effect was seen on the expression levels of STAT5A (Figure 5C, 7).

The same experiments were performed on CD33^+^ cells isolated from the bone marrows of patients with MDS (MDS BM-CD33). Figure 6 shows that the effects vorinostat (5 µM/8 h) on the expression of the same panels of genes described above in MDS BM-CD33 cells was comparable to that observed in PB-CD33 cells from AML patients (Figure 5). Exceptions were p15 and RAI3 that were not up-regulated in cells from all MDS patients; Gravin and STAT5A that were down-regulated in cells from all MDS patients; and Cyclin D2, CDK4, and CDK6 which were down-regulated in most MDS patients cells as compared to AML PB-CD33 cells.

Therefore, the overall effect of vorinostat on the expression of apoptosis genes in primary PB and BM CD33^+^ cells is pro-apoptotic and similar to that seen in THP1 but less marked than that observed in K562 and HL60 despite different profiles of gene expression.

In summary, vorinostat alters the profile of expression of genes involved in proliferation, apoptosis and differentiation leading to a pattern that would predict inhibition of cell proliferation and increase in differentiation. This is consistent with its functional effect on haematopoietic cell lines and primary cells.

### Vorinostat Responsive Promoter Elements of IER3, cFOS and COX2 Genes are Located within their Proximal Promoter Regions

To explore the molecular mechanism by which vorinostat modulates gene expression, reporter plasmids containing different portions of the promoter regions of IER3, cFOS and COX2, some of those genes which we found to be most responsive to vorinostat, were generated and used in reporter assays in K562 and HL60. The luciferase activity of the full length IER3, cFOS and COX2 reporter plasmids was significantly enhanced by treatment with vorinostat (Figure 7A and Figure S3). Deletions of the IER3 promoter sequence between −2000 and −163 slightly reduced the promoter activity in K562 (Figure 7A) and HL60 (data not shown) but had no significant effect on the vorinostat-mediated enhancement. However, deletion of the sequence −91 to −61 abolished both vorinostat-mediated and basal promoter activities. Results obtained with cFOS and COX2 reporter plasmids indicate their vorinostat-responsive elements are located in the −449/+155 region of cFOS and downstream of −246 of COX2 (Figure S3).

The −91/−61 region of IER3 contains binding sites for the transcription factors CP2, AP2, AP4, MZF1 and SP1 (Figure 7B). The putative TF binding sites in this region were ablated individually by site-directed mutagenesis to investigate whether they could be responsible for its basal and vorinostat-mediated activity (Figure 7B). The transcriptional activity of these mutated constructs in K562 and HL60 cells indicate that the putative SP1/MZF1 DNA binding site present in the −72/−65 region is an essential regulator of IER3 basal expression and that it is necessary for the vorinostat-enhanced expression of this gene in these cells (Figure 7C).

### Vorinostat Transcriptional Effects are Regulated by Zinc Finger TF

**Figure 8:**
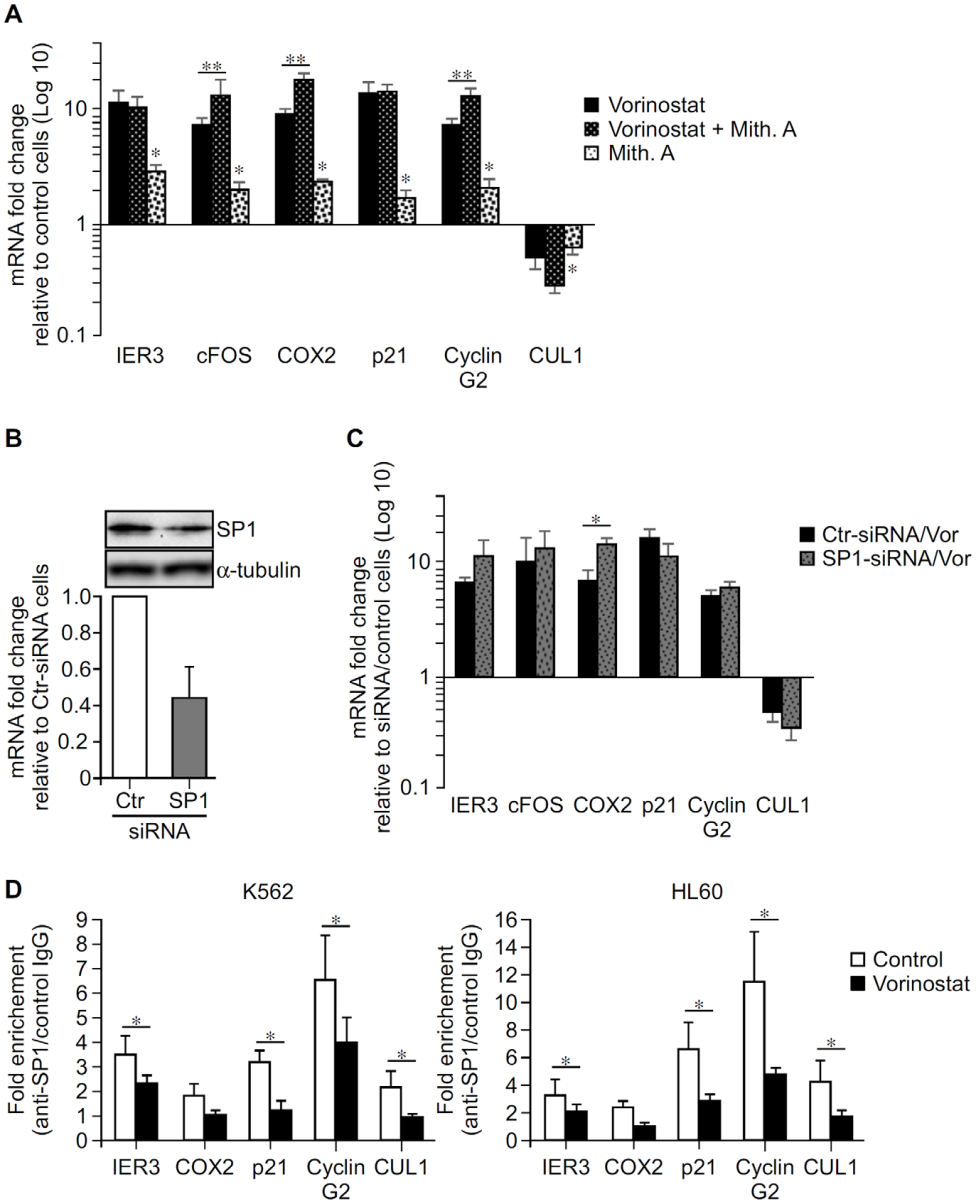
Effect of GC-rich DNA sequences and SP1 on the transcriptional effect of vorinostat on IER3, cFOX, COX2, p21, Cyclin G2 and CUL1 genes. A, K562 cells were exposed to Mith.A (100 nM) or vehicle (Control) and not further treated or exposed 30 min later to 5 µM vorinostat for 8 h and gene expression quantified by qPCR. Results are average values ± SEM of at least three independent assays, done in triplicate. B–C, K562 cells were transfected with control (Ctr) or SP1 siRNA and treated with vehicle or vorinostat (2 µM) for 24 and 48 h. B upper panel, immunoblot showing SP1 protein levels in cells transfected with control (Ctr) and SP1 siRNAs in 1 out of 5 assays at 48 h after transfection. B lower panel, SP1 mRNA levels in cells transfected with control (Ctr) and SP1 siRNAs. Graph shows average percentage of SP1 mRNA ± S.D. from 5 independent assays at 24 h after transfection. C, Graph shows average mRNA fold change of the indicated genes over vehicle treated cells transfected with the same siRNA 48 h after transfection ± SEM from 3 independent experiments. D, K562 and HL60 cells were treated with vorinostat (5 µM) or vehicle (Control) for 7 h and SP1 binding to the proximal promoter regions of the indicated genes determined by ChIP assays and qPCR. Results are expressed as fold change over control IgG and represent average values of at least three independent experiments ± SEM. Data were analyzed using paired Student’s t test. *p<0.05. **p<0.05.

Bioinformatic analysis of the proximal promoter regions of IER3, cFOS, COX2, p21, Cyclin G2 and CUL1 revealed GC-rich DNA sequences to which zinc finger TFs (SP1, MZF1, ER-alpha, MAZ, ETF) can bind (Figure S4), including motifs similar to the GGGAGG sequence identified in IER3 (Figure 7B). Since the transcriptional effect of HDACi on some genes depends on TF that bind to proximal GC-rich DNA sequences, especially SP1 [19], [20], [21], [44], we explored whether the transcriptional effect of vorinostat one these genes could be regulated by zinc finger TF binding to GC-rich DNA sequences. To do so, we tested whether Mith.A, a potent inhibitor of binding of zinc finger TF, especially SP1 to GC-rich DNA sequences, which selectively interfere with SP1-mediated gene transcription [25], [26], [49], [50], would affect the transcriptional action of vorinostat. Inhibition of zinc finger TF binding to GC-rich DNA sequences with Mith.A in K562 significantly potentiated the transcriptional effect of vorinostat on cFOS, COX2 and Cyclin G2 but not on IER3, p21 and CUL1 (Figure 8A). In addition, Mith.A alone up-regulated IER3, cFOS, COX2, p21, and Cyclin G2 and down-regulated CUL1 in a fashion similar to but less marked than vorinostat. These results suggest the involvement of GC-rich DNA sequences in basal transcription of these genes and in the vorinostat action in the expression of cFOS, COX2 and Cyclin G2.

To explore the involvement of SP1 in vorinostat-mediated gene expression, we assessed the effects of SP1 knockdown via RNAi. Transfection of K562 with siRNA against SP1 resulted only in a maximum of 65% decrease in SP1 mRNA and protein levels, as compared to control siRNA (Figure 8B). Nonetheless, SP1 reduction by 30–50% with SP1 specific siRNA increased by 2 fold the transcriptional effect of vorinostat on COX2 (Figure 8C), as compared to cells transfected with control siRNA. No consistent effects of such amounts of SP1 reduction were observed in vorinostat-mediated expression of IER3, cFOS, p21, Cyclin G2, and CUL1 genes between different experiments.

### Vorinostat Modulates Binding of SP1 to Gene Proximal Promoter Regions

SP1 can bind to the GC-rich DNA sequence shown to be essential for vorinostat-mediated expression of IER3 (Figure 7B and C) as well as to the GC-rich DNA sequences located in the promoter regions of cFOS, COX2, p21, Cyclin G2 and CUL1 (Figure S4). ChIP assays were performed to determine whether SP1 binds to the proximal promoter regions of these genes in haematopoietic K562 and HL60 cells and whether vorinostat affects its binding. In the absence of vorinostat, SP1 bound to the proximal promoters of IER3, COX2, p21, Cyclin G2 and CUL1. Vorinostat treatment led to significant reduction of this binding in both cells (Figure 8D).

## Discussion

There is increasing evidence that, in addition to genetic mutations, epigenetic events play a critical role in the pathophysiology of haematopoietic disorders such as MDS and AML. Accordingly epigenetic agents, i.e. hypometylating agents such as azacytidine and decitabine, and HDACi like vorinostat and romidepsin have been tested and shown to produce responses in myeloid disorders both *in vitro* and *in vivo* [2], [3], [5], [6], [7], [8]. Clinical studies revealed not all AML and MDS patients respond to vorinostat [10], [13], and the precise mechanisms of action of vorinostat on AML and MDS cells remain poorly understood. The identification of its functional effects and potential target genes may contribute to the identification of markers that predict response to vorinostat, which would be extremely useful to identify those patients most likely to respond to vorinostat. In this study, we therefore investigated the functional and transcriptional effects of vorinostat in MDS and AML, using human promyelocytic HL60 (AML M2/3), monocytic THP1 (AML M5), and erythroleukaemic K562 (cells with AML M6 properties) cell lines as an *in vitro* model and primary myeloid cells from AML and MDS patients to confirm our initial findings.

Functional assays revealed vorinostat promoted cell cycle arrest, and induced apoptosis, growth inhibition and differentiation of HL60, K562, and THP1 cells (Figures 1 and 2 and Figures S1 and S2). Importantly, differentiation of HL60, K562 and THP1 cell lines was observed at concentrations of vorinostat that induced significant growth inhibition and apoptosis, suggesting these events are associated. However, intermediate monocytic differentiation of THP1 was observed at vorinostat concentrations that caused cell growth inhibition but not apoptosis. HL60 and THP1 were more sensitive to cell cycle arrest, growth inhibition and differentiation induced by vorinostat than K562. This observation might suggest that the efficacy of vorinostat in promoting cellular differentiation depends on its effect on promoting cell cycle arrest and growth inhibition. This remains however to be established. Amongst the three cell lines, THP1 was most sensitive to the apoptotic effect of vorinostat and K562 least sensitive to its induction of cell cycle arrest, growth inhibition, apoptosis and differentiation.

Importantly, vorinostat also promoted myeloid differentiation of CD33^+^ cells from MDS patients and patients with AML M2, M4 and M5 types (Table 1 and Figure 3). Myeloid differentiation of these cells was also associated with increased apoptosis, once again suggesting these events are correlated.

The profile of gene expression produced by vorinostat in HL60, K562, THP1 and primary myeloid cells is consistent with the promotion of cell cycle arrest, growth inhibition, differentiation and apoptosis of the neoplastic cells. In our experiments, vorinostat modulated the expression of cell cycle, apoptosis, and differentiation genes known to be altered in haematologic malignancies, increasing expression of those genes normally down-regulated in MDS and/or AML and suppressing genes normally over-expressed in these malignancies (Figures 4, 5, 6). Vorinostat up-regulated the expression of genes involved in cell signalling (cFOS, RAI3) [7], [8], cell cycle arrest (p15, p21, Cyclin G2) [6], [40], [44], cell cycle checkpoint and DNA repair (RAD9A) [45], cell differentiation (NOTCH2) [51] and down-regulated the expression levels of important genes involved in cell cycle transition (Cyclin D1, CUL1) [5], [7], [8], double strand break repair (NBN), cell proliferation and survival (c-MYC, AXL, MZF1, STAT5A, NFκB1, TNFRSF8) [7], [30], [38], [39], [52], [53], [54]. Pro-apoptotic genes (e.g. IER3, p21, PPP1R13B and caspase activators) [7], [8], [55] are those preferentially up-regulated (Figures 4, 5, 6).

The gene expression pattern produced by vorinostat suggest it arrested K562 cells at the G1 and G2/M phases probably by inducing p21 and Cyclin G2 and repressing cell cycle transition genes CUL1, Cyclin D2, and CDK4. HL60 cells were arrested at the G2/M phase probably through increased expression of Cyclin D1 and p21, which are involved in cell cycle G1/S transition and cell cycle arrest at G2/M phase, respectively. THP1 were arrested at the G1 phase likely due to up-regulation of cell cycle arrest genes p21 and Cyclin G2, and down-regulation of cell cycle transition gene CDK6.

The patterns of expression of apoptosis genes generated by vorinostat suggest HL60 cells were more sensitive to vorinostat-induced apoptosis than K562 cells probably due to higher expression levels of pro-apoptotic genes p21 and CD40 (Figures 4B, 5 and 4C, 6, respectively), fewer suppressed pro-apoptotic genes (two versus four in Figure 4C, 6) and lower induction of anti-apoptotic genes (Figure 4C, 7). Interestingly, though vorinostat induced less pro-apoptotic genes and caused lower modulation of these genes in THP1 than in HL60 and K562 cells, THP1 cells were substantially more sensitive to the apoptotic effects of vorinostat. This might be attributed to substantially lower induction of the caspase inhibitor genes IFI6 and SERPINB9 in these cells, as compared to HL60 and K562. Another explanation might be that THP1 apoptosis by vorinostat also relies on the modulation of other apoptosis genes and/or non-transcriptional effects of vorinostat such as generation of reactive oxygen species or modulation of protein activity. This last notion is supported by published data showing vorinostat changes cellular function via multiple mechanisms of action [15]. This hypothesis remains to be clarified.

Interestingly, the different pro-apoptotic gene expression patterns in response to vorinostat amongst AML PB-CD33 and MDS BM-CD33 cells and leukemic K562, HL60 and THP1 cells (Figs 4, 5, 6) suggests vorinostat promoted apoptosis of these cells via different molecular mechanisms. In primary cells, mechanisms involving the FOXL2 and IFT57 caspase activators are likely to be at play whereas in cell lines the mechanisms probably depend on p21, CD40, and HIP1 proteins. Moreover, our results showing that vorinostat–mediated down-regulation of cell cycle transition genes was more marked in MDS than in AML cells (Figures 5 and 6), and that up-regulation of the cell cycle arresting gene p15 was greater in AML than in MDS cells suggest that vorinostat promotes cell cycle arrest of these primary myeloid cells through different molecular mechanisms. In MDS BM-CD33 cells these mechanisms involve the Cyclin D2, CDK4 and CDK6 proteins, whereas in AML cells the mechanisms are dependent on p15. This hypothesis is supported by published data showing p15 is one of the genes most frequently inactivated in leukaemic patients by DNA methylation and that p15 methylation in AML patients is associated with poor prognosis [40], [41].

Our findings showing vorinostat induced terminal differentiation of HL60 but only partial erythroid differentiation of K562 (Figure 2 and Figure S2) suggest vorinostat causes differentiation in a cell line specific manner. Since K562 cells can undergo terminal erythroid differentiation by other HDACi (e.g. apicidin) [56], the different effects of vorinostat in HL60 and K562 differentiation might partially result from the different gene expression profiles (e.g. p15, c-MYC, apoptosis genes) in response to vorinostat amongst K562 and HL60 cells (Figure 4). Our results showing vorinostat promoted monocytic terminal differentiation of only a very small proportion of THP1 cells (less than 6% in Figure 2L and N) may be explained by the high rate of apoptosis at the vorinostat concentrations required for terminal differentiation.

Overall, the effects of vorinostat in growth inhibition and differentiation of malignant haematopoietic cells shown herein may be, at least partially, mediated through the modulation of the expression of genes that control proliferation, apoptosis and differentiation such as cFOS, COX2, IER3, p21, p15, RAI3, Cyclin D1, c-MYC, AXL and MZF1 [7], [8], [30], [38], [39], [42]. The importance of p21 up-regulation in the vorinostat functional effects is supported by its important role in controlling cell proliferation, apoptosis and differentiation and data showing the p21 gene is activated by most of the tested HDAC inhibitors, suggesting that p21 might in part mediate the antiproliferation and differentiation effects of these drugs [44], [57]. The importance of c-MYC down-regulation is supported by its important role in haematopoiesis/leukaemogenesis and data showing that its down-regulation is critical for valproic acid induced growth arrest and myeloid differentiation of AML cells [39], [58].

Analysis of the promoter regions of IER3, cFOS and COX2, some of the most responsive genes, identified vorinostat responsive elements within proximal promoter regions (Figure 7A and Figure S3). Subsequent ablating mutation of a putative SP1/MZF1 binding site within the proximal IER promoter markedly reduced basal and abrogated vorinostat-induced promoter activity (Figure 7B and C). Analysis of proximal IER3, cFOS, COX2, p21, Cyclin G2 and CUL1 promoter sequences showed they contain GC-rich DNA sequences to which SP1 and other zinc finger TF can bind, including similar motifs to the SP1/MZF1 present in the IER3 promoter (Figure S4). Furthermore, inhibition of transcriptional activity mediated by GC-rich DNA sequences with Mith.A, a compound that binds to GC-rich DNA sequences and interferes with zinc finger TF binding, especially SP1 [49], [50], potentiated the effects of vorinostat on the expression of cFOS, COX2 and Cyclin G2, and changed basal expression of IER3, cFOS, COX2, p21, Cyclin G2 and CUL1 in the same fashion as vorinostat though to a much less extent (Figure 8A). In addition, 30–50% reduction of SP1 with specific siRNA increased vorinostat-induced COX2 expression by 2 fold (Figure 8B–C). Finally, ChIP assays showed SP1 binds to the proximal promoter regions of all these genes except cFOS and that vorinostat decreased its binding to IER3, p21, Cyclin G2 and CUL1 (Figure 8D). SP1 binding to proximal COX2 region was lower as compared with other genes but consistently decreased by vorinostat.

Although these findings do not show direct evidence for the involvement of SP1 via GC-rich DNA elements in the transcriptional modulation of these genes by vorinostat, they clearly demonstrate that vorinostat-mediated IER3 transcription relies on GC-rich DNA sequences located in its proximal promoter, and point to a role of these regulatory regions and zinc finger TF, e.g. SP1, in the transcriptional effects of vorinostat in these genes [49], [50]. Namely, that GC-rich DNA sequences are involved in vorinostat-mediated expression of IER3, cFOS, COX2 and Cyclin G2. Also they suggest that the transcriptional effect of vorinostat in IER3, COX2, p21, Cyclin G2, and CUL1 might have occurred through a mechanism dependent on the dissociation of SP1 from their proximal promoters, which cannot be attributed to its down-regulation by vorinostat (Figure 4A, 3). Whether the transcriptional modulation by vorinostat relies on SP1 dissociation from GC-rich DNA sequences remains however to be established. The observations that i) ablation of the SP1/MZF1 site within the proximal IER3 promoter decreased basal and impaired vorinostat-induced transcription and ii) vorinostat decreased SP1 binding to proximal IER3 region, suggest that vorinostat either disrupted SP1 binding from a downstream SP1 consensus motif (Figure S4) or that vorinostat disrupted SP1 binding from this site allowing the recruitment of others zinc finger TFs that acted as transcriptional activators of IER3 in the absence of SP1. Furthermore, the fact that Mith.A potentiated vorinostat-induction of cFOS expression and no SP1 binding to its proximal promoter was found indicates that vorinostat-mediated transcription of this gene is probably dependent on other zinc finger TFs.

Our results are in conformity with increasing data showing that gene-modulation by HDACi (vorinostat, butyrate, TSA, apicidin, valproic acid) is mediated via SP1 motifs through SP family TFs [18], [20], [59]. Transcriptional activation or repression by SP1 depends on the promoter context it binds to and on the co-activators and co-repressors it interacts with. These interactions and direct binding competition between SP1 and other zinc finger TFs are important in the transcriptional regulation of genes with GC-rich DNA sequences located proximal to transcription initiation site. SP1 has been shown to repress transcription of several genes including p21 by recruiting HDAC and co-repressors complexes such as NCo-R, SMRT, and NuRD, to their proximal promoters [18], [21], [60]. Accordingly, HDACi-mediated expression of these genes acted via disruption of SP1 binding from their promoters [18], [20], [59]. For some genes, e.g. Cyclin G2, SP1-dependent recruitment of HDAC and co-repressors involved SP1 interaction with other zinc finger TF such as ER-alpha at the GC-rich motifs in its proximal promoter region [61], [62]. Our results are also consistent with recent data showing that acetylation of SP1 by HDACi decreases its DNA binding affinity allowing binding of weaker affinity zinc finger TFs, e.g. SP3, to the same site which can act as transcriptional activators or repressors [19], [20].

Whether modulation of MZF1 expression (Figure 4A, 3) and/or activity by vorinostat accounts for the transcriptional effects of vorinostat on these genes is a question that remains to be elucidated. However, since MZF1 can repress transcription via recruitment of HDAC to gene promoters [63] and some of these genes have putative binding sites for MZF1 (Figure S4), it is plausible that MZF1 may play a role in vorinostat-mediated transcriptional regulation of some of these genes.

In conclusion, these results identify new vorinostat-responsive genes in leukemic cells and, most important, in primary myeloid cells from AML and MDS patients, some of them often deregulated in these malignancies, and implicated in their pathogenesis, and point to a strong correlation between the functional and transcriptional effects of vorinostat. Moreover, they show IER3 transcription by vorinostat is mediated by proximal promoter GC-rich DNA sequences, and suggest regulation by GC-rich DNA sequences and SP1 are involved in vorinostat action in some of these genes. Confirmation of this data in the course of clinical trials might shed further light of the usefulness of this drug in the treatment of myeloid disorders.

## Supporting Information

Figure S1 **Effect of vorinostat on cell cycle progression and apoptosis of K562 and HL60 cells.** A-B, K562 cells were cultured with 5 µM vorinostat or vehicle (Control) and cell cycle distribution analyzed 24 h thereafter by flow cytometry. A, Average percentage of K562 cells in each phase of the cell cycle ± SD of three independent assays, done in triplicate. B, Representative histograms obtained in K562 showing the effect of vorinostat in K562 cell cycle progression. Cells arrested in the G1 phase, 2 N DNA content; cells arrested in S phase; and cells arrested in the G2/M phase (4 N DNA content). C–D, K562 and HL60 cells were treated with vorinostat or vehicle (Control) as indicated. After 72 h, apoptosis was determined by flow cytometry. C, Average percentage of apoptotic K562 and HL60 cells ± SD of three independent experiments, done in duplicate. D, Representative dot blots showing the percentage of apoptotic K562 and HL60 cells cultured for 72 h in the absence and in the presence of vorinostat. Numbers are percentage of total cells in the respective gates. *p<0.05.
(TIF)

Figure S2 **Effect of vorinostat on terminal erythroid differentiation of K562 cells.** K562 cells were treated with vorinostat or vehicle (Control) as indicated. After 4 days, terminal differentiation of K562 was examined by measuring Hb content by ELISA and by microscopy of benzidine (to detect Hb) plus Giemsa stained cells. A, Quantification of hemoglobin content in K562 cells cultured in the presence of vorinostat and vehicle from two different assays, each done in triplicate. Results are expressed as nanograms of Hb per micrograms of total cellular protein ± SD (n = 3) in two independent assays. B, benzidine-Giemsa stain of K562 cultured in the absence and in the presence of 2 µM vorinostat during 4 days from two independent assays. Similar results were obtained in K562 cells after 3 and 5 days in culture in the absence and presence of vorinostat.
(TIF)

Figure S3 **Identification of vorinostat responsive elements in the cFOS and COX2 promoters.** A, K562 and HL60 cells were transiently co-transfected with pGL3-bascic vector or reporter constructs containing different DNA sequences of the cFOS promoter cloned into the pGL3-luciferase reporter along with β-galactosidase control vector as indicated. 1 h after transfection the cells were treated with 2 µM vorinostat or vehicle (Control). Cell lysates were obtained 24 h after and assayed for luciferase and β-galactosidase activities. Luciferase activities were normalized to β-galactosidase units in the same samples. B, K562 and HL60 cells were transiently co-transfected with pGL3-bascic vector or reporter constructs containing different DNA sequences of the COX2 promoter cloned into the pGL3-luciferase reporter along with β-galactosidase control vector as indicated and the rest of the procedure was done as in (A). Results in (A and B) are average fold induction ± S.D *versus* control cells transfected with pGL3-basic of one of three independent assays, done in triplicate, using each reporter plasmid at least from two different clones. Data were analyzed using the ANOVA and the Tukey-Kramer multiple comparison test. *p<0.05.
(TIF)

Figure S4 **Scheme of the proximal promoter regions of IER3, COX2, cFOS, p21, Cyclin G2 and CUL1 genes.** Promoter regions of indicated genes were analyzed for the presence of TF binding sites by using the online Transcription Element Search System. The putative binding sites for SP1 and other zinc finger transcription factors present in these sequences are shown. Motifs identical or similar to the GGGAGG motif present in IER3 −71/−66 promoter region, which is crucial to its basal and vorinostat-mediated expression as by reporter assays, are highlighted.
(TIF)
