# Supplementary figures and images for: Vorinostat Induces Apoptosis and Differentiation in Myeloid Malignancies: Genetic and Molecular Mechanisms
Source: PLoS One. 2013 Jan 8;8(1):e53766. doi: 10.1371/journal.pone.0053766 (PMC3540071; doi:10.1371/journal.pone.0053766)

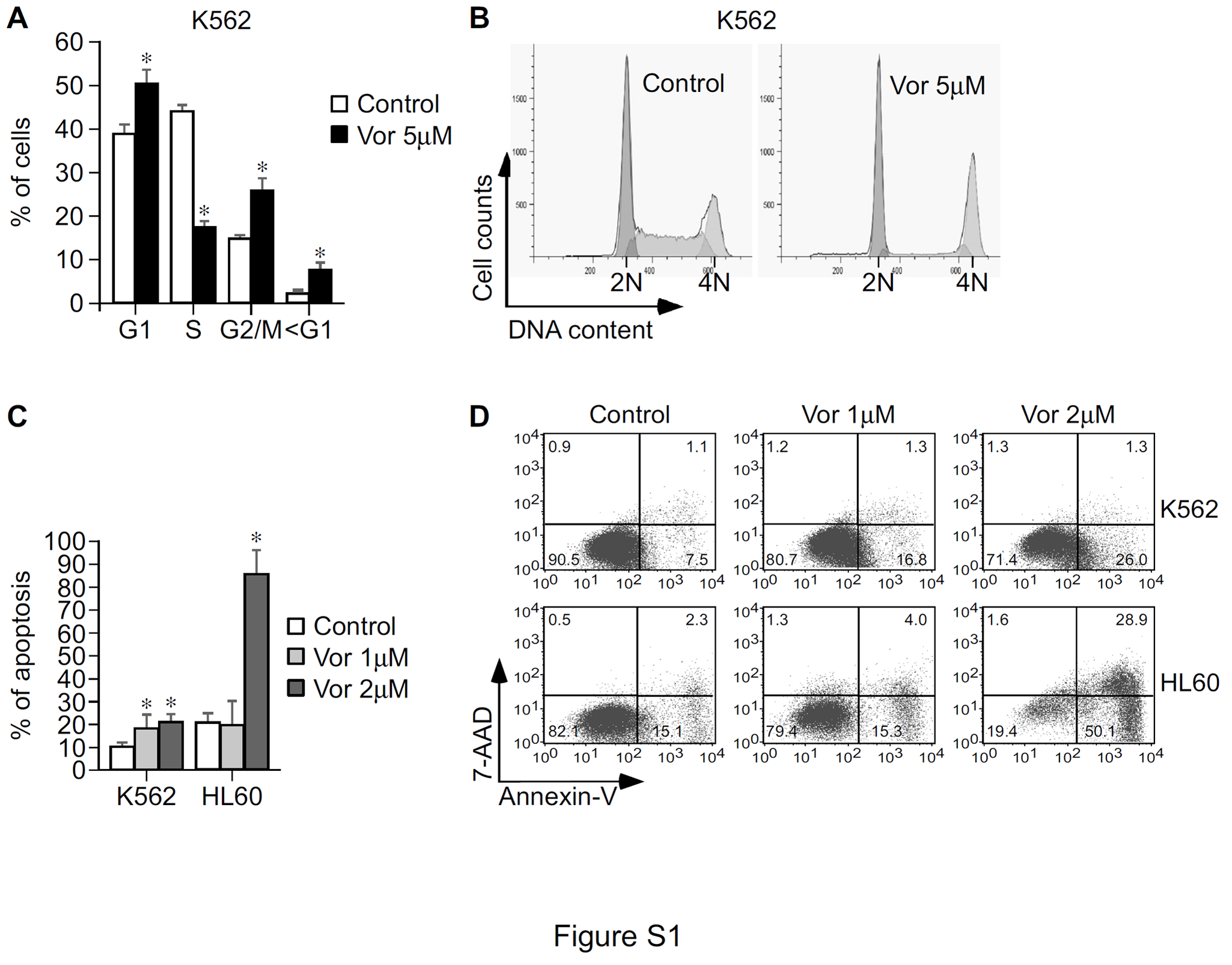

Supplement: Figure S1 — Effect of vorinostat on cell cycle progression and apoptosis of K562 and HL60 cells. A-B, K562 cells were cultured with 5 µM vorinostat or vehicle (Control) and cell cycle distribution analyzed 24 h thereafter by flow cytometry. A, Average percentage of K562 cells in each phase of the cell cycle ± SD of three independent assays, done in triplicate. B, Representative histograms obtained in K562 showing the effect of vorinostat in K562 cell cycle progression. Cells arrested in the G1 phase, 2 N DNA content; cells arrested in S phase; and cells arrested in the G2/M phase (4 N DNA content). C–D, K562 and HL60 cells were treated with vorinostat or vehicle (Control) as indicated. After 72 h, apoptosis was determined by flow cytometry. C, Average percentage of apoptotic K562 and HL60 cells ± SD of three independent experiments, done in duplicate. D, Representative dot blots showing the percentage of apoptotic K562 and HL60 cells cultured for 72 h in the absence and in the presence of vorinostat. Numbers are percentage of total cells in the respective gates. *p<0.05. (TIF) [file pone.0053766.s001.tif]

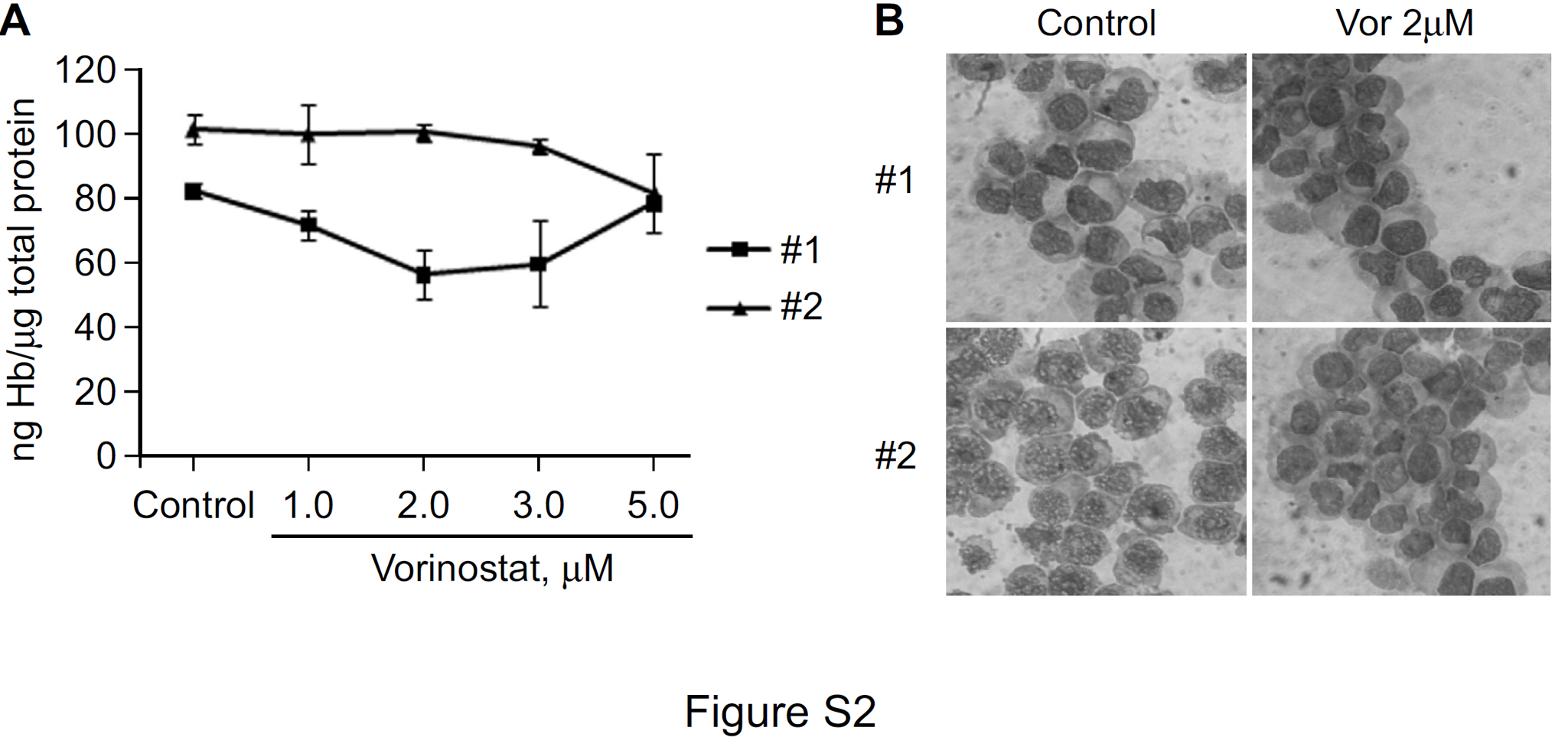

Supplement: Figure S2 — Effect of vorinostat on terminal erythroid differentiation of K562 cells. K562 cells were treated with vorinostat or vehicle (Control) as indicated. After 4 days, terminal differentiation of K562 was examined by measuring Hb content by ELISA and by microscopy of benzidine (to detect Hb) plus Giemsa stained cells. A, Quantification of hemoglobin content in K562 cells cultured in the presence of vorinostat and vehicle from two different assays, each done in triplicate. Results are expressed as nanograms of Hb per micrograms of total cellular protein ± SD (n = 3) in two independent assays. B, benzidine-Giemsa stain of K562 cultured in the absence and in the presence of 2 µM vorinostat during 4 days from two independent assays. Similar results were obtained in K562 cells after 3 and 5 days in culture in the absence and presence of vorinostat. (TIF) [file pone.0053766.s002.tif]

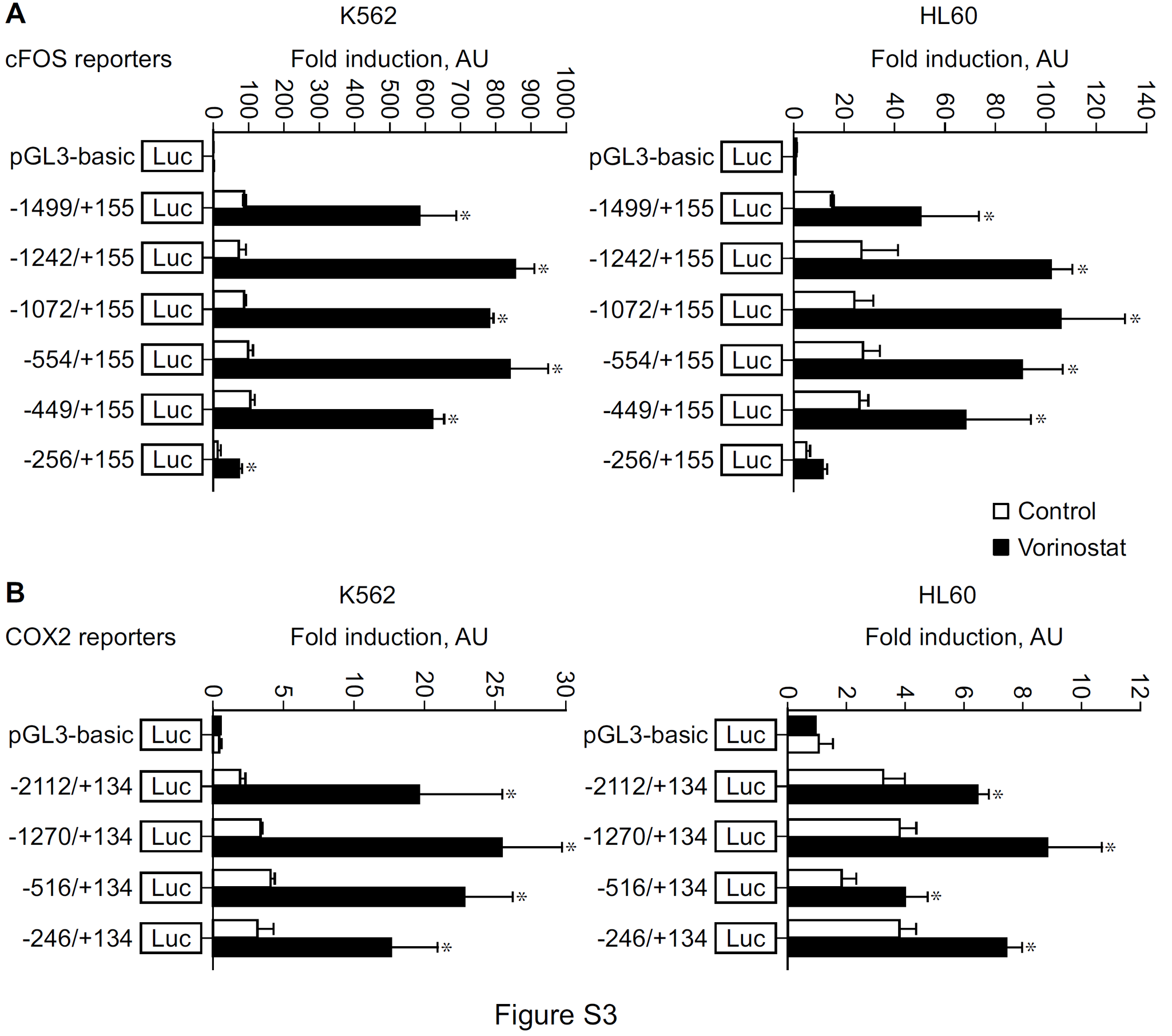

Supplement: Figure S3 — Identification of vorinostat responsive elements in the cFOS and COX2 promoters. A, K562 and HL60 cells were transiently co-transfected with pGL3-bascic vector or reporter constructs containing different DNA sequences of the cFOS promoter cloned into the pGL3-luciferase reporter along with β-galactosidase control vector as indicated. 1 h after transfection the cells were treated with 2 µM vorinostat or vehicle (Control). Cell lysates were obtained 24 h after and assayed for luciferase and β-galactosidase activities. Luciferase activities were normalized to β-galactosidase units in the same samples. B, K562 and HL60 cells were transiently co-transfected with pGL3-bascic vector or reporter constructs containing different DNA sequences of the COX2 promoter cloned into the pGL3-luciferase reporter along with β-galactosidase control vector as indicated and the rest of the procedure was done as in (A). Results in (A and B) are average fold induction ± S.D versus control cells transfected with pGL3-basic of one of three independent assays, done in triplicate, using each reporter plasmid at least from two different clones. Data were analyzed using the ANOVA and the Tukey-Kramer multiple comparison test. *p<0.05. (TIF) [file pone.0053766.s003.tif]

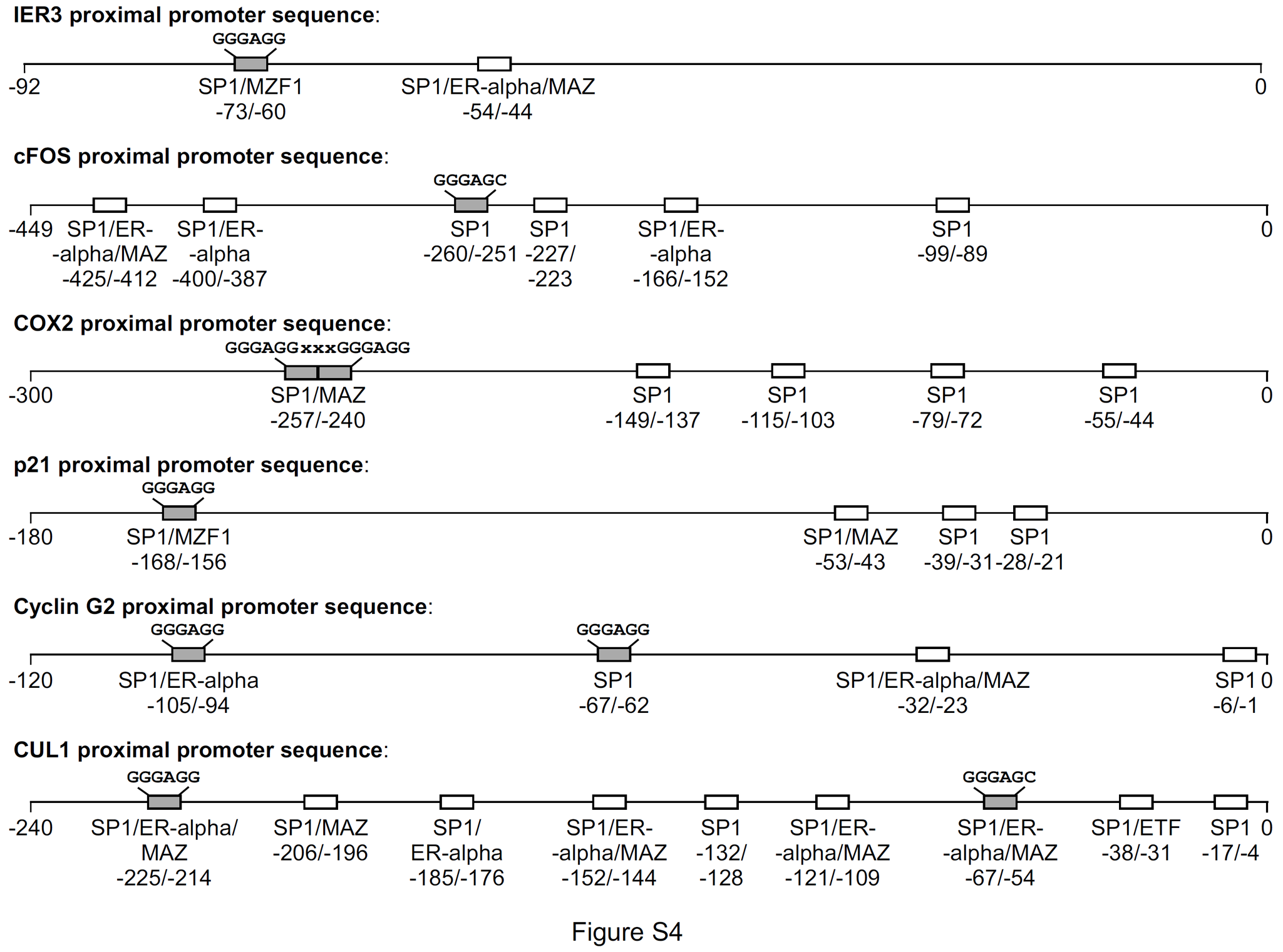

Supplement: Figure S4 — Scheme of the proximal promoter regions of IER3, COX2, cFOS, p21, Cyclin G2 and CUL1 genes. Promoter regions of indicated genes were analyzed for the presence of TF binding sites by using the online Transcription Element Search System. The putative binding sites for SP1 and other zinc finger transcription factors present in these sequences are shown. Motifs identical or similar to the GGGAGG motif present in IER3 −71/−66 promoter region, which is crucial to its basal and vorinostat-mediated expression as by reporter assays, are highlighted. (TIF) [file pone.0053766.s004.tif]
